# Neonatal Maternal Separation Modifies Proteostasis Marker Expression in the Adult Hippocampus

**DOI:** 10.3389/fnmol.2021.661993

**Published:** 2021-07-22

**Authors:** Jorge A. Sierra-Fonseca, Jameel N. Hamdan, Alexis A. Cohen, Sonia M. Cardenas, Sigifredo Saucedo, Gabriel A. Lodoza, Kristin L. Gosselink

**Affiliations:** ^1^Department of Biological Sciences and Border Biomedical Research Center, University of Texas at El Paso, El Paso, TX, United States; ^2^Neuroscience Program, Smith College, Northampton, MA, United States; ^3^Department of Physiology and Pathology, Burrell College of Osteopathic Medicine, Las Cruces, NM, United States

**Keywords:** autophagy, mitophagy, proteasome, sex differences, aging, stress, early life adversity

## Abstract

Exposure to early-life stress (ELS) can persistently modify neuronal circuits and functions, and contribute to the expression of misfolded and aggregated proteins that are hallmarks of several neurodegenerative diseases. The healthy brain is able to clear dysfunctional proteins through the ubiquitin-proteasome system (UPS) and the autophagy-lysosomal pathway (ALP). Accumulating evidence indicates that impairment of these pathways contributes to enhanced protein aggregation and neurodegeneration. While stress is a known precipitant of neurological decline, few specific mechanistic links underlying this relationship have been identified. We hypothesized that neonatal maternal separation (MatSep), a well-established model of ELS, has the ability to alter the levels of UPS and ALP components in the brain, and thus has the potential to disrupt proteostasis. The expression of proteostasis-associated protein markers was evaluated by immunoblotting in the hippocampus and cortex of adult Wistar rats that were previously subjected to MatSep. We observed multiple sex- and MatSep-specific changes in the expression of proteins in the ALP, mitophagy, and UPS pathways, particularly in the hippocampus of adult animals. In contrast, MatSep had limited influence on proteostasis marker expression in the cortex of adult animals. Our results indicate that MatSep can selectively modify the intracellular protein degradation machinery in ways that may impact the development and progression of neurodegenerative disease.

## Introduction

Early life stress (ELS) has been demonstrated to exert profound physiological effects with long-lasting consequences on human health (Lai and Huang, 2011). Accumulating evidence indicates that adverse early life events such as maternal deprivation, malnutrition, abuse, neglect, and loss of a parent can increase vulnerability to a variety of pathological conditions including cardiovascular disease, obesity, anxiety, and neuropsychiatric disorders later in life (Heim et al., 2004; Taylor, 2010; Hoeijmakers et al., 2015; Chen and Baram, 2016). Epidemiological data suggest that children exposed to ELS are at higher risk for a variety of mental health problems, with dysfunction of the hypothalamic-pituitary-adrenal axis implicated as a critical underlying cause of these outcomes (Halligan et al., 2007; Essex et al., 2011; Trickett et al., 2011). Furthermore, it is becoming increasingly clear that ELS can influence the later development of neurodegenerative diseases. ELS is capable of altering HPA axis function, enhancing its reactivity, and increasing the levels of circulating glucocorticoids which can subsequently alter dendritic morphology and cause synaptic changes. This, in turn, has been associated with increased brain vulnerability to neurodegenerative processes (Esch et al., 2002; Brunson et al., 2005; Oomen et al., 2010; Hoeijmakers et al., 2018). Although this neuroendocrine imbalance is known to be present in patients with neurodegenerative conditions, such as Alzheimer’s and Parkinson’s diseases (Huang et al., 2009; Ros-Bernal et al., 2011), the specific mechanisms that underlie the highly complex ELS-neurodegeneration relationship are still poorly understood. However epigenetic modifications (Stankiewicz et al., 2013; Mpofana et al., 2016), changes in neuronal gene expression (Bravo et al., 2014; Zimmer and Spencer, 2014), neuroinflammation (Ganguly and Brenhouse, 2014), and abnormal protein accumulation in neuronal cells (Martisova et al., 2013; Lesuis et al., 2018) have emerged as candidates. Here, we have more thoroughly investigated the possible contributions of abnormal protein aggregation by examining the effects of neonatal maternal separation (MatSep), a model of ELS, on the expression of protein markers of proteostasis in the brain.

Proteostasis involves a network of highly sophisticated cellular mechanisms that are responsible for protein quality control, including synthesis, folding, modification, trafficking, degradation, and recycling (Balch et al., 2008; Labbadia and Morimoto, 2015). Even under normal physiological conditions, up to 30% of newly synthesized proteins are misfolded or prone to aggregation, therefore requiring consistent proteostasis network function to maintain the integrity of the proteome (Princiotta et al., 2003; Dunker et al., 2008). Components of the proteostasis network include two main protein degradation systems: autophagy and the ubiquitin-proteasome system (UPS; Tanaka and Matsuda, 2014; Díaz-Villanueva et al., 2015).

**Figure 1:**
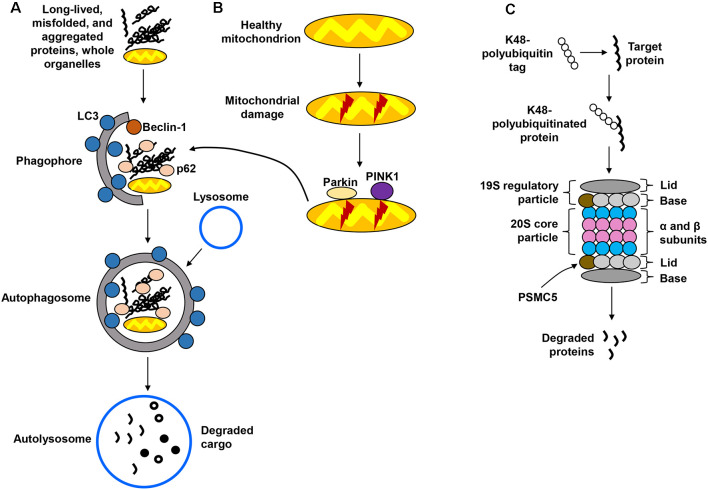
Protein degradation pathways. **(A)** The autophagy-lysosomal pathway (ALP) is in charge of degrading long-lived and misfolded and aggregated proteins, as well as whole organelles. Components targeted for degradation are engulfed in autophagosomes, which are ultimately fused with the lysosome for cargo degradation. **(B)** Mitochondria are also degraded via the lysosome in a specialized form of autophagy called mitophagy, which recognizes damaged mitochondria through protein sensors (PINK1, parkin) that target the organelles for disposal via the ALP. **(C)** The ubiquitin-proteasome system (UPS) employs ubiquitin tags to target proteins for degradation through the 26S proteasome. Several steps in these pathways have been omitted for simplification.

Macroautophagy, hereafter referred to as the autophagy-lysosomal pathway (ALP; Figure 1A) is the better-characterized form of autophagy and consists of a lysosome-coupled bulk degradation process that involves the formation of double-membrane vesicles known as autophagosomes (Shen and Mizushima, 2014; Kiriyama and Nochi, 2015). These vesicles start as isolation membranes known as phagophores, which then expand to engulf multiple intracellular components including proteins, organelles, and protein aggregates. This cargo then becomes enclosed inside the now-formed autophagosome, which is trafficked and fused with the lysosome to undergo degradation. The resulting products are then recycled for future cellular use (Sarkar et al., 2007; Yang and Klionsky, 2009). Although the ALP was once believed to function by randomly degrading cellular materials, it has now become clear that it is a highly selective process capable of targeting specific components, including organelles such as mitochondria. The autophagic removal of damaged mitochondria is referred to as mitophagy (Figure 1B) and occurs through a complex signaling system that involves the actions of several molecules, including parkin, an E3 ubiquitin ligase, and PTEN-induced putative kinase 1 (PINK1; Narendra et al., 2008; Jin et al., 2010; Fedorowicz et al., 2014). The UPS (Figure 1C), distinct from but complementary to the ALP, targets soluble, short-lived proteins through ubiquitin tagging as a signal for proteolytic degradation in the proteasome (Goldberg, 2003; Finley, 2009). Ubiquitin is covalently linked to a lysine residue of the target protein through a series of enzymatic reactions, with the attached ubiquitin molecule potentially being itself a target for multiple ubiquitination cycles, therefore giving rise to polyubiquitin chains (Ravid and Hochtrasser, 2008; Vilchez et al., 2014). Modification of proteins by polyubiquitination at lysine 48 (K48) serves as a highly specific signal for degradation via the 26S proteasome (Komander, 2009; Saez and Vilchez, 2014). The 26S proteasome is a large, multicatalytic molecular complex (~2.5 MDa in size) comprised of a catalytic core particle, the 20S proteasome formed by multiple α and β subunits with structural and catalytic functions, respectively (Groll et al., 1997; Beck et al., 2012; Vilchez et al., 2014), and two 19S regulatory subunits that participate in the recognition of proteins targeted for degradation (Smith et al., 2007; Lander et al., 2012; Schreiber and Peter, 2014).

Impairment of the neuronal proteostasis network, particularly the protein degradation machinery, is linked to a variety of pathological conditions and neurological disorders (Hipp et al., 2014; Henning and Brundel, 2017). Multiple factors are known to affect the proteolytic capacity of the proteostasis network, including mutations, aging, and cellular and physiological stress (Lim and Yue, 2015; Xu et al., 2016; Henning and Brundel, 2017; Korovila et al., 2017). Evidence provided by rodent models indicates that stress is able to alter autophagic and proteasomal processes in the brain. Male rats subjected to chronic unpredictable mild stress were found to display elevated hippocampal autophagic activity (Hou et al., 2015), and restraint stress has been shown to disrupt the expression of autophagic, mitophagic, and proteasomal markers in the rat brain (Orlovsky et al., 2014; Jevtić et al., 2016). Physical exercise has also been shown to influence the autophagic and mitophagic pathways in the rat brain (He et al., 2012; Marques-Aleixo et al., 2015). While these animal studies successfully demonstrate that diverse stress paradigms can impact proteostasis function, the stressors were applied in adult animals, and therefore do not address the potential effects that stress experienced during the early life period can exert on proteostasis. In rodents, neonatal MatSep is a well-established model for the study of ELS. MatSep is known to influence brain development, induce long–lasting neurochemical changes, alter neuroimmunological and stress responses, and enhance vulnerability to a variety of neurological disorders such as depression, addiction, and neurodegeneration (Aisa et al., 2007; Nishi et al., 2014; Gracia-Rubio et al., 2016; Hui et al., 2017; Grigoruta et al., 2020). A previous study showed that MatSep can influence autophagy in adult rat hippocampus and prefrontal cortex (Liu et al., 2018), and another study demonstrated that levels of FKBP5, a co-chaperone protein involved in proteostasis, can modulate the effects of MatSep on anxiety-like behavior in adult mice (Criado-Marrero et al., 2019), thus supporting the notion that this ELS paradigm has the ability to modify proteostasis responses later in life. Given this evidence, the present study aimed to determine if MatSep, a widely validated model for the study of ELS, can alter the protein degradation machinery, specifically the autophagic/mitophagic and proteasomal systems, in the adult rat brain. Using immunoblotting analysis, we assessed the impacts of neonatal MatSep on the protein degradation machinery by measuring the expression of specific protein markers belonging to the ALP, mitophagy, and UPS pathways in the hippocampus and cortex of both male and female rats, with additional attention given to aged rat populations. To the best of our knowledge, this is the first study to investigate the effects of MatSep on the expression of proteostasis markers in two distinct brain regions, while also examining the contributions of sex and age.

## Materials and Methods

### Experimental Animals

Wistar rats were bred in-house and housed (two per cage) in standard cages in a temperature- and humidity-controlled animal facility maintained on a 12:12 h light cycle with food and water available *ad libitum*. All animals were cared for in accordance with the Guide for the Care and Use of Laboratory Animals, and all procedures were approved by the Institutional Animal Care and Use Committee (IACUC protocol #A-201006–1).

### Early Life Stress

Neonatal Wistar rats were subjected to MatSep according to previously described protocols (Huot et al., 2002; Kalinichev et al., 2002), with some modifications (Grigoruta et al., 2020). During post-natal days (PND) 2–14, dams and pups were removed from their home cage for 3 h daily beginning at 0800 h. Dams were placed in a holding cage under an extraction hood, while the pups were moved to a different cage containing surgical bedding and placed on top of a circulating water bath set at 37°C. Once the separation period was over, dams and pups were returned to their home cage. Control animals were not separated from their dams and were only gently and briefly handled daily to mimic the transfer of the MatSep pups between cages. After the completion of the MatSep protocol, all rats were left undisturbed except for weekly cage changes. Pups were weaned on PND 21, pair-housed under the conditions described above, and allowed to grow to adulthood (~3 months old) or more advanced age (~16 months old) before experimental use. Experimental animals were grouped as follows: Control adult females (*n* = 6) and males (*n* = 6), as well as adult MatSep females (*n* = 4) and males (*n* = 6); aged Control females (*n* = 5) and males (*n* = 5), and aged MatSep females (*n* = 5).

### Brain Tissue Harvest and Homogenization

Rats were lethally anesthetized with an intraperitoneal injection of sodium pentobarbital (50 mg/mL; Akorn Pharmaceuticals, Lake Forest, IL). The brain was immediately dissected from the skull, quickly frozen on dry ice, and stored at −80°C until use. Brain tissue was thawed on ice and the hippocampus and a dorsal region of the cortex were dissected and homogenized manually with a Teflon pestle hand-held glass homogenizer using T-PER buffer supplemented with protease and phosphatase inhibitor tablets (Thermo Fisher Scientific, Rockford, IL). Homogenates were cleared by centrifugation at 10,000× *g* for 10 min, and supernatants saved for Western blotting. Protein concentrations in the homogenates were determined using the Pierce bicinchoninic acid assay (Thermo Fisher Scientific, Rockford, IL) with bovine serum albumin (BSA) serving as a standard.

### Antibodies

A list of primary antibodies and concentrations used in this study is provided in Supplementary Table 1. Alkaline phosphatase (AP)-conjugated secondary antibodies (goat anti-rabbit IgG and goat anti-mouse IgG) were purchased from Southern Biotech (Birmingham, AL).

### Western Blotting

Equal amounts (25 μg) of total hippocampal and cortical proteins were subjected to SDS-PAGE using 12.5% pre-cast gels (BioRad, Hercules, CA), followed by protein electrotransfer onto PVDF membranes. The membranes were blocked in a buffer containing 5% BSA dissolved in TBS (100 mm Tris-HCl and 150 mm NaCl, pH 7.4) for 1 h at room temperature, followed by overnight incubation at 4°C with primary antibody dissolved in blocking buffer (see Supplementary Table 1 for primary antibody details). The membranes were then washed with TBS-T (TBS with 0.05% Tween 20) and incubated with the appropriate AP-conjugated secondary antibodies (1:2,000 dilution in blocking buffer) for 1 h at room temperature. For sensitive detection of the protein bands, Immun-Star chemiluminescent reagent (BioRad, Hercules, CA) was employed. When differences in molecular weight allowed, more than one protein was detected in the same membrane (e.g., LC3-II and p62). All membranes were subsequently washed with TBS-T and stripped by conducting three washes of 5 min at room temperature in mild stripping buffer (200 mm glycine, 0.1% SDS, and 1% Tween 20, pH 2.2), followed by extensive washing with TBS-T. Membranes were then re-probed with actin antibody (1:2,000 dilution in blocking buffer) for loading control. Immunoblotting experiments were conducted in triplicate for each individual marker. Densitometric analysis of the protein bands for each marker was conducted using the LabWorks software (UVP laboratory products, Upland, CA), and these values were subsequently normalized to total actin.

### Statistical Analysis and Data Presentation

All statistical analyses were conducted using Sigma Plot 12.5 software (Systat, Chicago, IL). Samples from all animals included in the study were analyzed by immunoblotting for every protein marker. Outlier data points were identified and excluded from analysis if they were found to be ≥1.5 times greater than the interquartile range. When comparing the protein expression levels between experimental groups of adult animals, overall effects of the significant factors of sex (male vs. female) and treatment (Control vs. MatSep) were assessed using two-way analysis of variance (ANOVA), followed by Bonferroni *post hoc* analysis to account for comparing between multiple experimental groups. When comparing between adult and aged animals, a one-way ANOVA with Bonferroni *post hoc* analysis was used, with age as the significant factor. No comparisons were made across brain regions. In all cases, a value of *p* ≤ 0.05 was considered to be statistically significant. Densitometric values displayed in the graphics represent the mean ± standard error of the mean (SEM) of all available samples (after excluding outliers), corresponding to data points obtained from at least three independent blots.

## Results

In the present study, Western blotting was employed to determine changes in protein expression in the brains of adult rats that had been previously exposed to MatSep in the neonatal period. We assessed the levels of protein markers involved in three different intracellular protein degradation pathways, the ALP, mitophagy, and the UPS, in hippocampal and cortical tissue. Differential effects were analyzed using two-way ANOVA followed by Bonferroni *post hoc* analysis, with treatment (Control, MatSep) and sex as independent variables for all comparisons involving adult animals. We also assessed whether sex differences or MatSep-induced changes were observed in aged animals, utilizing one-way ANOVA with Bonferroni *post hoc* analysis. Importantly, the experimental group containing male aged MatSep rats was excluded from the present study, given the limited number of animals available for analysis. For the entire datasets containing average densitometric values for each marker, see Supplementary Materials (Supplementary Tables 2, 3). For statistical analyses, including *p*, *F*, and *t* values, as well as degrees of freedom, see Supplementary Tables 4–7.

### MatSep Increases Autophagy Marker Expression in the Hippocampus, but Not the Cortex, of Adult Rats

**Figure 2:**
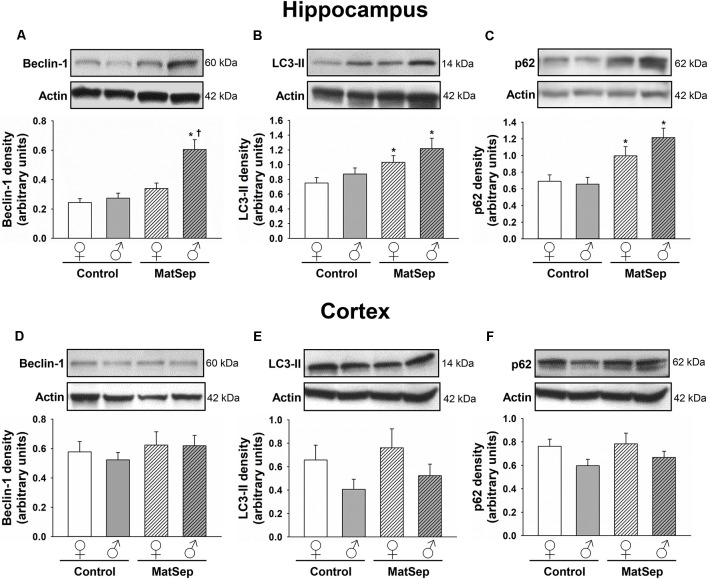
Immunoblot analysis of ALP markers from hippocampal and cortical homogenates from adult rats subjected to MatSep. Panels above each graph show representative blots for **(A,D)** beclin-1, **(B,E)** LC3-II, and **(C,F)** p62. Bar graphs represent densitometric analysis of protein expression normalized to actin and averaged over at least three separate blots. *p ≤ 0.05 vs. same-sex Controls; ^†^*p* ≤ 0.05 when comparing between sexes.

The effects of MatSep on autophagy were assessed through Western blotting analysis of ALP markers in hippocampal (Figures 2A–C) and cortical (Figures 2D–F) tissues from adult rats subjected to MatSep. Three specific markers were selected: beclin-1, which participates in autophagy initiation and autophagosome formation; the phospholipid-conjugated form of LC3 (LC3-II), which is recruited to autophagosomes; and p62, the prototypical autophagy receptor (Pankiv et al., 2007; Kang et al., 2011; Lamb et al., 2013). Two-way ANOVA showed that hippocampal expression of the three autophagy markers was increased by MatSep when compared to Controls. For beclin-1 (Figure 2A), two-way ANOVA showed significant main effects of MatSep (*F* = 10.368, *p* ≤ 0.05) and sex (*F* = 21.266, *p* ≤ 0.05), with the interaction between both factors also showing significance (*F* = 6.562, *p* ≤ 0.05) on the expression of this autophagy initiator. Specific group effects include a significant MatSep-induced increase in beclin-1 expression in males (*t* = 5.416, *p* ≤ 0.05), as well as a sex difference in MatSep animals (*t* = 3.815, *p* ≤ 0.05), as the increased expression of beclin-1 was not observed in females. MatSep had an overall significant effect on LC3-II expression in the hippocampus, as demonstrated by two-way ANOVA (Figure 2B; *F* = 10.364, *p* ≤ 0.05), but no significant effects were observed for the sex variable or the MatSep-sex interaction (*p* > 0.05). Accordingly, *post hoc* analysis showed increased LC3-II levels in both females (*t* = 2.002, *p* ≤ 0.05) and males (*t* = 2.562, *p* ≤ 0.05). For p62 expression, only a significant MatSep main effect was observed (Figure 2C; *F* = 19.868, *p* ≤ 0.05), and Bonferroni *post hoc* analysis showed that both sexes displayed significant increases in p62 compared to their respective same-sex Controls (*t* = 2.116, *p* ≤ 0.05 for females; *t* = 4.321, *p* ≤ 0.05 for males). No overall nor specific group effects of MatSep were observed on the expression of any of the three ALP markers in adult cortical tissue (Figures 2D–F; *p* > 0.05), except for a significant overall effect of sex for p62 (Figure 2F; *F* = 4.383, *p* ≤ 0.05) in this region.

### Abnormal Expression of Mitophagy Markers is Seen Following MatSep in the Adult Hippocampus

**Figure 3:**
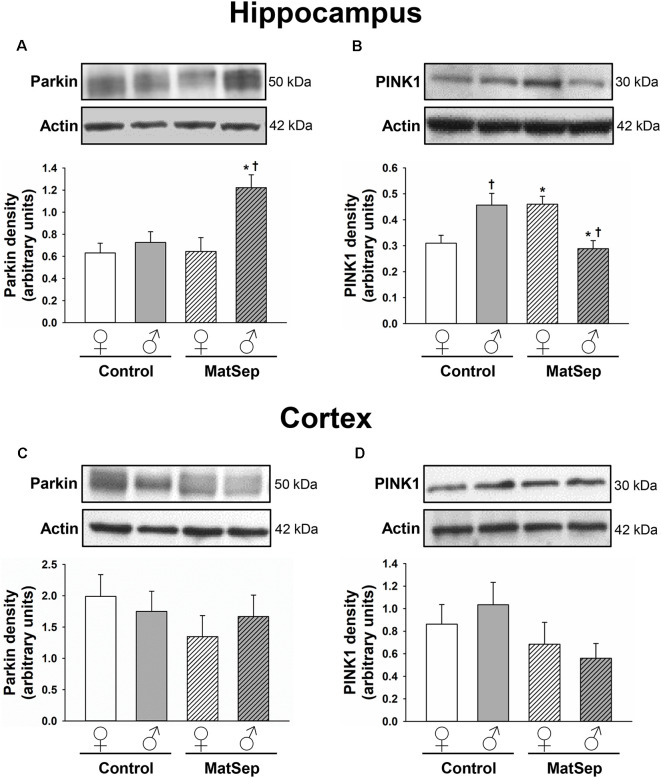
Immunoblot analysis of mitophagy proteins from hippocampal and cortical homogenates from adult rats subjected to MatSep. Panels above each graph show representative blots for **(A,C)** parkin and **(B,D)** PINK1. Bar graphs represent densitometric analysis of protein expression normalized to actin and averaged over at least three separate blots. *p ≤ 0.05 vs. same-sex Controls; ^†^*p* ≤ 0.05 when comparing between sexes.

To determine the influence of MatSep on the expression of mitophagy markers in the adult brain, we measured the levels of two mitophagic proteins by Western blotting. Hippocampal (Figures 3A,B) and cortical (Figures 3C,D) homogenates were evaluated for their levels of parkin, which serves as a signal for mitophagy initiation, and PINK1, a serine/threonine kinase that acts as a sensor for mitochondrial damage and is responsible for recruiting parkin to the mitochondria (Narendra et al., 2008; Jin et al., 2010). In the case of parkin expression (Figure 3A), two-way ANOVA revealed significant main effects of MatSep (*F* = 17.885, *p* ≤ 0.05) and sex (*F* = 5.426, *p* ≤ 0.05), as well as an interaction between these two factors (*F* = 4.837, *p* ≤ 0.05). Bonferroni *post hoc* analysis showed that parkin expression in the adult male hippocampus was significantly elevated by MatSep compared to Control males (Figure 3A; *t* = 3.494, *p* ≤ 0.05), with a significant sex difference due to the fact that a similar increase was not seen in females (Figure 3A; *t* = 3.535, *p* ≤ 0.05). For PINK1 expression (Figure 3B), no overall effects of MatSep or sex were observed (*p* > 0.05), although a significant interaction of both variables was detected (*F* = 17.885, *p* ≤ 0.05). Bonferroni *post hoc* analysis revealed that when compared with Control animals of the same sex, MatSep females displayed increased PINK1 expression (*t* = 2.673, *p* ≤ 0.05) while MatSep males showed decreased PINK1 expression (*t* = 3.359, *p* ≤ 0.05) in this region. Significant sex differences were also observed in hippocampal PINK1 expression, in both Control (*t* = 3.065, *p* ≤ 0.05) and MatSep animals (*t* = 2.953, *p* ≤ 0.05; Figure 3B). No significant differences were found in the cortical expression of parkin or PINK1 in response to MatSep (Figures 3C,D; *p* > 0.05).

### UPS Marker Expression Is Modified by MatSep in the Hippocampus and Cortex of Adult Rats

**Figure 4:**
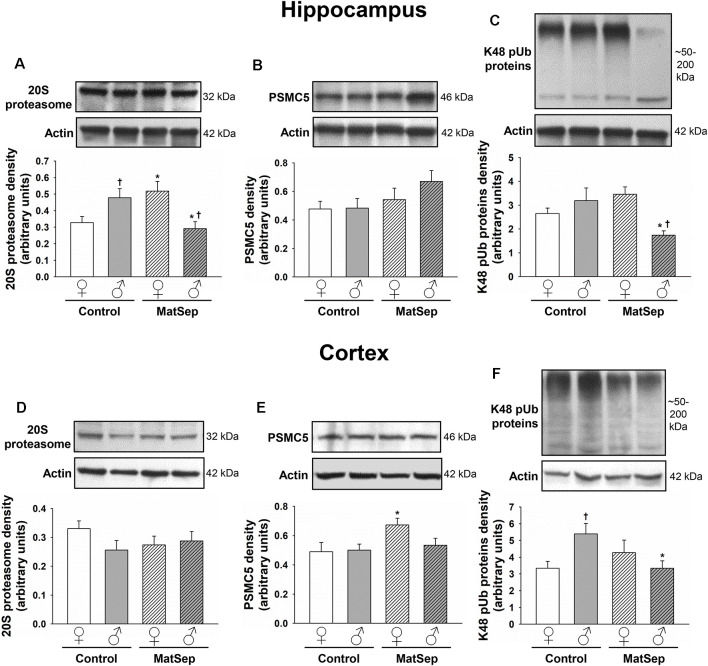
Immunoblot analysis of proteasomal markers from hippocampal and cortical homogenates from adult rats subjected to MatSep. Panels above each graph show representative blots for **(A,D)** 20S proteasome, **(B,E)** PSMC5, and **(C,F)** K48 pUb proteins. Bar graphs represent densitometric analysis of protein expression normalized to actin and averaged over at least three separate blots. *p ≤ 0.05 vs. same-sex Controls; ^†^*p* ≤ 0.05 when comparing between sexes.

To determine the effects of MatSep on the UPS, we employed antibodies against three different components of this system: the 20S proteasome, which constitutes the catalytic core; PSMC5, which represents the 19S regulatory subunit; and K48-linked polyubiquitinated (K48 pUb) proteins, which represent proteins tagged with polyubiquitin chains that are targeted specifically for proteasomal degradation (Coux et al., 1996; Hochstrasser, 2000; Rechsteiner and Hill, 2005). Hippocampal (Figures 4A–C) and cortical (Figures 4D–F) regions of the brain were analyzed in adult female and male rats. In the adult hippocampus, no main effects of MatSep or sex (*p* > 0.05) were observed on 20S proteasome expression. However, a significant overall interaction of MatSep and sex was seen on the expression of this proteasomal marker, as demonstrated by two-way ANOVA (Figure 4A; *F* = 14.888, *p* ≤ 0.05). *Post hoc* analysis showed both MatSep- and sex-specific effects, with MatSep inducing a significant increase in 20S proteasome expression in females (*t* = 2.808, *p* ≤ 0.05) but decreasing the expression of this marker in the male hippocampus (*t* = 2.653, *p* ≤ 0.05). Furthermore, sex differences in 20S proteasome expression were apparent in the hippocampus of both Control (*t* = 2.298, *p* ≤ 0.05) and MatSep (*t* = 3.123, *p* ≤ 0.05) animals. PSMC5 expression in the adult hippocampus was not significantly altered by MatSep, and did not differ by sex (Figure 4B; *p* > 0.05). The expression of K48 pUb proteins, in contrast, was changed and showed a significant MatSep–sex interaction in this effect (Figure 4C; *F* = 9.842, *p* ≤ 0.05). Bonferroni *post hoc* tests further demonstrated that MatSep decreased the expression of K48 pUb proteins in the male, but not female, hippocampus (*t* = 3.040, *p* ≤ 0.05), with a significant sex difference also seen between the male and female MatSep groups (*t* = 3.196, *p* ≤ 0.05).

No MatSep- or sex-related changes were observed in cortical tissue for the 20S proteasomal marker (Figure 4D; *p* > 0.05). In contrast, two-way ANOVA revealed a significant main effect of MatSep on the expression of PSMC5 (Figure 4E; *F* = 4.204, *p* ≤ 0.05), with *post hoc* analysis indicating a MatSep-induced increase in PSMC5 in females (*t* = 2.05, *p* ≤ 0.05) with no such response in males (*p* > 0.05). Lastly, a significant interaction between sex and MatSep was detected for K48 pUb proteins in the cortex (Figure 4F; *F* = 7.292;* p* ≤ 0.05). Subsequent Bonferroni testing revealed significantly higher levels of K48 pUb proteins in males compared to female Control animals (*t* = 2.729, *p* ≤ 0.05), with a significant decrease in K48 pUb protein expression after MatSep in males only (*t* = 2.771, *p* ≤ 0.05).

### Effect of MatSep on the Levels of Proteostasis Markers in Aging Hippocampus and Cortex

**Figure 5:**
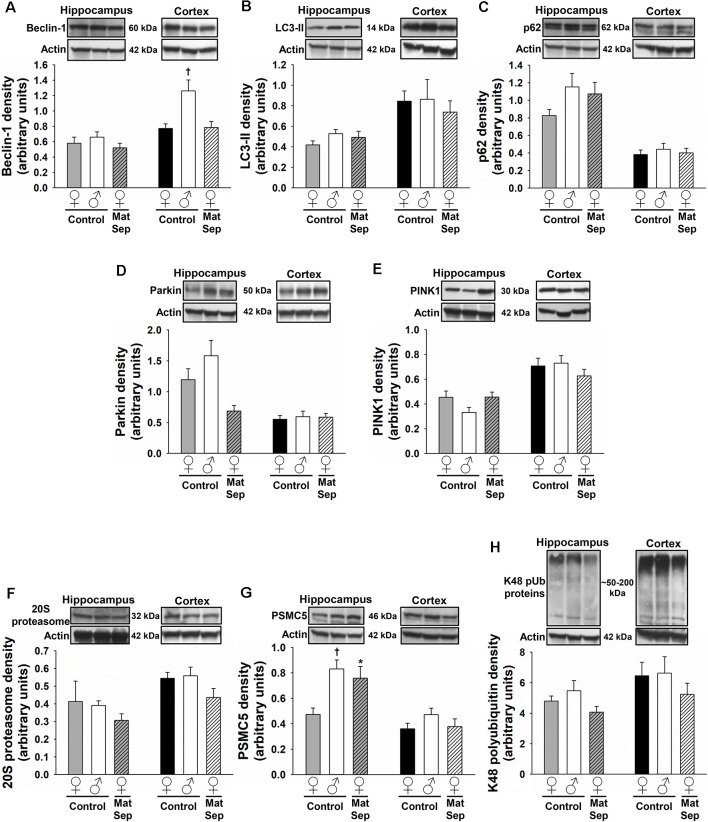
Immunoblot analysis of proteostasis markers from hippocampal (gray-white bars) and cortical (black-white bars) homogenates from aged rats. Panels above each graph show representative blots depicting the ALP markers beclin-1 **(A)**, LC3-II **(B)**, and p62 **(C)**; the mitophagic proteins parkin **(D)** and PINK1 **(E)**; and the UPS markers 20S proteasome **(F)**, PSMC5 **(G)**, and K48 pUb proteins **(H)**. Actin was used to normalize protein band density over at least three separate blots in all cases. *p ≤ 0.05 vs. same-sex Controls; ^†^*p* ≤ 0.05 when comparing between sexes.

To examine whether the effects of MatSep on protein degradation pathways persist, resolve, or are enhanced in the brains of aged animals, we assessed the levels of the same proteostasis markers in the hippocampus and cortex of 16-month old rats, including some that had been subjected to MatSep as neonates. As previously stated, only a small number of male MatSep aged animals were available for study, therefore this experimental group was excluded from the present analysis. Data from the remaining groups were analyzed by one-way ANOVA with Bonferroni correction as a *post hoc* test (Figure 5 and Supplementary Table 5).

In the evaluation of ALP markers, no effects of MatSep were observed in any of the tested markers in the aged rat hippocampus (Figures 5A–C, gray-white bars; *p* > 0.05). In the mitophagic markers from the aged hippocampus (Figures 5D–E, gray-white bars), we found that MatSep led to a significant main effect in parkin expression (*F* = 4.803, *p* ≤ 0.05) without any individual group effects, while no significant differences were observed for PINK1 expression (*p* > 0.05). The expression of 20S proteasome or K48 pUb proteins was not affected by MatSep in the aged hippocampus (Figures 5F–H, gray-white bars; *p* > 0.05). However, hippocampal PSMC5 expression (Figure 5G, gray-white bars) was significantly changed by the MatSep and sex interaction (*F* = 7.996, *p* ≤ 0.05), with increased PSMC5 levels seen in females (*t* = 2.936, *p* ≤ 0.05) and a significant sex difference observed in Control animals (*t* = 3.687, *p* ≤ 0.05).

In cortical samples, MatSep had no effect on the levels of LC3-II or p62 expression in aged female animals (Figures 5B,C, black-white bars; *p* > 0.05). Similarly, no MatSep effects were seen on the cortical expression of the mitophagic proteins or any of the UPS markers (Figures 5D–H, black-white bars; *p* > 0.05). For beclin-1 expression, however, a significant main effect was observed (Figure 5A, black-white bars; *F* = 7.607, *p* ≤ 0.05), with a significant sex difference seen in aged Control beclin-1 expression (*t* = 3.356, *p* ≤ 0.05).

### Effects of MatSep and Age on the Expression of Proteostasis Markers: Differences Between Adult and Aged Animals

As an extension of our data analysis, we conducted a comparative assessment of the expression levels of all proteostasis markers in aged compared to adult animals (see Supplementary Tables 6, 7 for complete statistical analysis). First, we focused on differences resulting from the interaction of the significant factors of age and MatSep, which informs the possibility that MatSep may induce alterations in proteostasis marker expression in younger adult animals that are typically observed in advanced age. Secondly, we assessed the effect of age independently from MatSep. That is, differences in proteostasis marker expression caused only by advanced age compared to adult animals. To address our first point of interest, we compared adult MatSep groups with Control aged animals. A lack of statistical difference (*p* > 0.05) would suggest that the levels of proteostasis markers in adult MatSep animals approximate those observed in Control animals of advanced age. Such a finding would indicate that MatSep can influence the molecular characteristics of the brain, leading to protein expression levels in younger animals that are more reminiscent of those typically observed in aging subjects. In hippocampal tissue from both male and female rats (see Supplementary Table 6 for statistical analysis), the expression of ALP markers beclin-1 and p62 in MatSep adult rats was similar to that in aged Control animals. Hippocampal LC3-II levels, in contrast, were significantly different between MatSep adults and aged Controls of both sexes. The expression of both mitophagic markers, parkin and PINK1, as well as the 20S proteasome and PSMC5 UPS markers, were similar in adult MatSep rats and aged Control animals, regardless of sex. A clear difference was seen between adult MatSep and aged Control males regarding the levels of K48 pUb protein expression in the hippocampus. This effect was specific to males and was not seen in adult MatSep compared to aged Control female rats; although this comparison did approach significance (*p* = 0.07). In the cortex (see Supplementary Table 7 for statistical analysis), ALP markers in adult MatSep animals with expression levels similar to those seen in aged Controls included beclin-1 in females, LC3-II in both sexes, and p62 in males. For the mitophagic proteins, parkin expression differed only in adult MatSep compared to aged Control males, while PINK1 levels were similar between aged Controls and adult MatSep animals of both sexes. Additional sex differences were observed in the expression of the UPS markers, with PSMC5 in males and K48 pUb proteins in females shown to be similar in adult MatSep animals compared to aged Controls.

Secondly, we evaluated differences in proteostasis marker expression that occur in response to aging alone by statistically comparing results from Control adult rats of both sexes with those from their aged Control counterparts. In hippocampal tissues (Supplementary Table 6), the expression of all ALP protein markers was significantly affected by age in one or both sexes. Beclin-1 expression was increased with age (*F* = 14.805, *p* ≤ 0.05) in both sexes (*t* = 4.574, *p* ≤ 0.05 for females and *t* = 4.823, *p* ≤ 0.05 for males). The opposite effect was seen for hippocampal LC3-II expression (*F* = 8.862, *p* ≤ 0.05), with significantly decreased levels of this marker seen in aged Control females (*t* = 3.460, *p* ≤ 0.05) and males (*t* = 3.204, *p* ≤ 0.05) compared to their adult Control counterparts. The levels of p62 expression were also significantly affected by age (*F* = 5.202, *p* ≤ 0.05), but only aged Control males showed significant increases in p62 expression compared to Control adult male animals (*t* = 3.607, *p* ≤ 0.05); this effect was not seen in females. The levels of the mitophagic marker parkin in the hippocampus were also increased significantly with advanced age (*F* = 7.828, *p* ≤ 0.05), and both sexes (*t* = 2.823, *p* ≤ 0.05 for females and *t* = 3.853, *p* ≤ 0.05 for males) displayed significant increases in expression, whereas the levels of the mitophagy sensor protein PINK1 were not significantly affected by age in either sex (*p* > 0.05). While the hippocampal levels of 20S proteasome were also unaffected by age in females or males (*p* > 0.05), PSMC5 expression was found to be increased in aged male animals only (*t* = 3.854, *p* ≤ 0.05). The levels of K48 pUb proteins in the aged hippocampus were significantly increased (*F* = 8.544, *p* ≤ 0.05), with both sexes displaying significant changes in this direction (*t* = 3.405, *p* ≤ 0.05 for females and *t* = 3.663, *p* ≤ 0.05 for males).

Age-related changes in the cortex (Supplementary Table 7) were fewer than those seen in the hippocampus. In males, the ALP markers of beclin-1 and LC3-II were both increased in aged compared to adult Control animals (*F* = 13.850 and 4.310, respectively, *p* ≤ 0.05). In contrast, the levels of p62 expression were significantly decreased in aged Control female cortex compared to adult Control females (*F* = 9.472, *p* ≤ 0.05). Parkin expression, one of the markers of mitophagy, was decreased in aged female and male Controls as compared to that observed in their same-sex adult Control groups (*F* = 12.158; *p* ≤ 0.05); no changes were seen in PINK1 expression. In both sexes, the expression of the 20 s proteasomal marker of the UPS was increased in aged Control vs. adult Control rats (*F* = 15.553; *p* ≤ 0.05). K48 pUb proteins, on the other hand, only demonstrated an age-related increase in expression in females (*F* = 4.058; *p* ≤ 0.05), and PSMC5 showed no changes in basal cortical expression with age in either sex.

## Discussion

ELS has long-term consequences on human health and is an established risk factor for the development of neuropsychiatric disorders (Schmidt, 2010). However, the cellular and molecular mechanisms underlying the effects of ELS on the brain remain largely unknown. The results presented here demonstrate that MatSep is sufficient to induce changes in the expression of multiple protein markers of the ALP, mitophagy, and UPS pathways, while also providing evidence that MatSep can induce differential effects based on sex, brain region, and age.

In order to obtain insights into the possible stages at which MatSep may disrupt ALP, we selected three markers that represent different steps along the autophagic pathway. First, we assessed the levels of beclin-1, an adaptor molecule critical for autophagosomal initiation and nucleation that is known to be highly expressed during autophagy (Meijer and Codogno, 2009; Nascimento-Ferreira et al., 2011). As revealed by immunoblotting analysis, beclin-1 expression was elevated after MatSep in the adult hippocampus, thus suggesting a potential increase in the initial steps of autophagy. Second, we analyzed levels of LC3-II, an autophagosomal protein known to be expressed in two different forms: the cytosolic LC3-I (16 kDa) and the phosphatidylethanolamine-conjugated LC3-II (14 kDa) which is found mainly in autophagosomes (Tanida et al., 2008). Given that LC3-II is associated with the autophagosomal membrane, its levels are generally accepted as a reliable marker of autophagy, with the LC3-II/LC3-I ratio also serving as a measure of autophagic activity (Mizushima and Yoshimori, 2007). The antibody employed in this study preferentially recognizes the LC3-II form, which was shown to be increased by MatSep in the adult hippocampus in both females and males. This finding appears to indicate an induction of autophagy by MatSep, given that levels of the autophagosomal form of LC3 are being increased. Our third marker, p62, is an autophagy receptor protein that functions as a selective autophagy target and is specifically incorporated into autophagosomes where it is subsequently degraded (Sahani et al., 2014). Levels of p62 immunoreactivity are therefore used as an indicator of autophagic flux, given that its levels decrease as the autophagy pathway successfully flows through to completion (Bjørkøy et al., 2005; Mizushima et al., 2010). Our data show that MatSep actually increases p62 levels in both adult and aged hippocampal tissues, therefore indicating incomplete autophagic flux (Komatsu et al., 2006; Pankiv et al., 2010). Together, our findings, therefore, indicate that, in the adult hippocampus, ELS can lead to enhanced autophagy initiation and autophagosome formation, given that both beclin-1 and LC3-II are increased after MatSep. On the other hand, elevated levels of p62 in our animals indicate a blockage of autophagy. This is consistent with previous studies showing that, under pathological conditions or chronic exposure to stressful insult, the autophagic machinery can become persistently blocked and insensitive to autophagic stimuli (Roesly et al., 2012; González-Rodríguez et al., 2014; Kim et al., 2017). We propose that ELS can alter the sensitivity of the ALP machinery and cause its functional impairment. ELS has also been established as an important contributor to mitochondrial dysfunction (Ghosh et al., 2016; Ridout et al., 2018), and our results further support these findings as we have demonstrated that our MatSep paradigm alters the expression of mitophagic proteins in both sexes and brain regions, in adult as well as in aged animals.

The results of the present study also indicate that the effects of ELS on the expression of proteostasis network markers are brain region-specific. The majority of the MatSep-induced changes we observed were in the hippocampus (see Table 1 for summarized results), with very few alterations seen in cortical tissue. This apparent hippocampal vulnerability to ELS is in line with a growing body of evidence which indicates that, as an important mediator in the stress response, the hippocampus is especially susceptible to the deleterious effects of insults such as ELS. The impacts of stress in the hippocampus can manifest as structural abnormalities, decreased plasticity, and altered neurogenesis, all of which may play a role in the later manifestation of neuropathology (Lee et al., 2001; Hanson et al., 2015; Hoeijmakers et al., 2015; Lajud and Torner, 2015). Furthermore, hippocampal damage resulting from the accumulation of abnormally aggregated proteins, which can occur as a result of impairing the process of proteostasis, is a hallmark of several neurodegenerative diseases (Moodley and Chan, 2014; Heckmann et al., 2020). While the cerebral cortex also plays a role in mediating the neurochemical stress response and is known to be functionally and structurally affected by ELS (Arnsten, 2009; Takatsuru and Koibuchi, 2015; Urb et al., 2019), experimental evidence indicates that it can be more resilient to stress (Takatsuru et al., 2009; Wang et al., 2014). Interestingly, a recent study involving adult male rats also found differential effects on autophagic activity by neuroanatomical region in response to MatSep, showing inhibition of the autophagy pathway in the hippocampus and activation in the prefrontal cortex (Liu et al., 2018). Our results thus confirm and extend these findings, and support the notion that ELS exerts distinct effects on proteostasis network pathways in the brain in a region-dependent manner. It is important to note, however, that the hippocampus and cortex are functionally connected and are therefore able to influence one another; these interactions may also be susceptible to the effects of stressors like MatSep (McEwen and Morrison, 2013).

**Table 1:** Significant changes in proteostasis marker expression that were seen in the hippocampus of adult rats exposed to MatSep.

Marker	Change with MatSep
Beclin-1	↑ in ♂
LC3-II	↑ in ♀ and ↑ in ♂
p62	↑ in ♀ and ↑ in ♂
Parkin	↑ in ♂
PINK1	↑ in ♀ and ↓ in ♂
20S proteasome	↑ in ♀ and ↓ in ♂
K48 pUb proteins	↓ in ♂

*↑, increased expression; ↓, decreased expression*.

Multiple sex-specific responses were detected in our study, in both the hippocampus and cortex and for many of the markers tested. Evidence from previous animal studies suggests that females are more susceptible to stress-related neuropathology, and while the underlying mechanisms of this susceptibility are not fully understood, some research efforts have focused on sex hormone alterations caused by ELS (McEwen and Morrison, 2013; Goodwill et al., 2018). Sex differences in autophagic and mitophagic activity have been reported in several cell types, including neuronal cells, with the interpretation that females display lower levels of basal autophagy but males are more sensitive to autophagy-induced cell death (Du et al., 2009; Campesi et al., 2013; Oliván et al., 2014; Weis et al., 2014; Demarest and McCarthy, 2015; Demarest et al., 2016). Sex differences have also been observed in the expression and activity of proteasomal complexes (Hansen et al., 2012; Rodriguez et al., 2014; Pomatto et al., 2017). Furthermore, at least in the case of autophagy, these sex differences appear to be established early in life, even during the prenatal period (Congdon, 2018). Given that sex differences in normal brain physiology as well as pathological states have been well established (Zagni et al., 2016; Ruszkiewicz et al., 2019), the results presented here serve to establish MatSep as a factor that can potentially predispose individuals to neurological disease later in life by impacting multiple components of the proteostasis network in a sex-specific manner.

An additional goal of our work was to determine if the effects of MatSep on the expression of proteostasis protein markers persisted from adulthood into later age. ELS has been shown to be a significant factor in the development of neuropathologies by negatively affecting neurogenesis, neuronal circuitry, and synaptic density (Teicher et al., 2006; Bath et al., 2016). Stress, particularly when experienced during the early life period, is emerging as a contributing factor to the appearance of neurodegenerative diseases, many of which only manifest with advanced age (Wilson et al., 2003; Hoeijmakers et al., 2018; Justice, 2018; Sierra-Fonseca and Gosselink, 2018). It is therefore critically important to detect and understand the ELS-induced damage on a cellular and molecular level that occurs before the onset of neuropathology. Our results show that MatSep can alter the levels of specific proteostasis markers in adulthood and in advanced age, in a sex- and brain region-specific manner. These findings are consistent with the notion of ELS negatively influencing later brain health, as has been previously demonstrated in both animal and human studies (Haapanen et al., 2018; Ruiz et al., 2018). Interestingly, most of the MatSep-induced changes in proteostasis marker expression that we observed in adult animals were not seen in aged animals. This apparent reversal seems to contradict the general notion that ELS negatively affects later neurological outcomes, or perhaps implies the existence of some compensatory mechanisms or saturation of the response to MatSep. The effects of ELS on the brain are known to be dependent on the timing and duration of the stress exposure, and can also be aggravated by further episodes of acute and chronic stress (Gee and Casey, 2015). Furthermore, ELS is known to affect the response to stressors that occur later in life (Maniam et al., 2014). Our experimental approach allowed us to explore MatSep-induced changes in the expression of specific proteostasis markers in adult animals, and examine whether similar changes persist in aged animals. Our model does not evaluate whether responses to additional acute stress stimuli are modified, nor does it recapitulate the condition of cumulative stress as our animals were not subject to additional stressors or other manipulations after the initial MatSep was applied. This could explain the absence of more prominent alterations in the expression of proteostasis markers in our aged MatSep animals. In addition, the reduced number of animals available limits our ability to detect other alterations or draw more solid conclusions from the experimental groups. Still, to the best of our knowledge, our study is the first to address the hypothesis that MatSep, a widely validated paradigm that models ELS, can differentially alter the expression of proteostasis markers with sex, brain region, and age specificity. Further studies will continue to inform the influence of MatSep and other stressors on proteostasis in the brain in males and females and with advanced age. The current findings also serve to illustrate the complex changes caused by MatSep that have the potential to affect specific protein degradation pathways in the proteostasis network by modifying the expression of individual components.

Chronic stress has been previously linked to accelerated aging on a cellular level (Price et al., 2013; Yegorov et al., 2020), with evidence also suggesting that ELS is strongly associated with premature aging and age-associated conditions, including neuropsychiatric disorders (Danese and McEwen, 2012; Zannas et al., 2015; Shalev and Belsky, 2016). In addition, decreased activity of the proteostasis network and the subsequent proteotoxicity that results from this alteration constitutes one of the cellular and molecular hallmarks of aging (López-Otín et al., 2013). In light of this evidence, we used our dataset to compare the expression of proteostasis markers in the hippocampus and cortex of adult rats that underwent MatSep as neonates, with those seen in animals of advanced age without previous MatSep exposure, that is, animals that were allowed to age normally. We did this in order to evaluate whether neonatal exposure to MatSep can modify proteostasis pathways in the brain of adult animals in such a way that they resemble a phenotype that would normally be seen in the aged brain, at least at the protein expression level. Our data show that the expression of multiple proteostasis markers in adult hippocampus and cortex from MatSep animals did not differ statistically (*p* > 0.05) from aged Control animals, suggesting that, as a result of neonatal MatSep, the expression of several components of the proteostasis network in adult animals is similar to what is typically seen in aged animals. Although preliminary, our findings potentially indicate that MatSep can promote molecular alterations consistent with advanced aging, a possibility that has not previously been explored and thus warrants further study. We also determined the impact of age on the expression of proteostasis markers independently of MatSep by statistically comparing Control groups of both adult and aged animals. Consistent with the notion of aging-induced alterations of proteostasis network components, our results show that expression levels of several markers from the ALP, mitophagy, and UPS pathways are affected solely by age and not MatSep, while also showing sex and region specificity. Interestingly, our results showed increased levels of K48 pUb proteins in both hippocampus and cortex with advanced age. Previous studies have demonstrated that the accumulation of ubiquitinated proteins increases with age, and this can result in neurotoxicity (Vernace et al., 2007; Klaips et al., 2018). Furthermore, in an attempt to clear these ubiquitinated proteins, cells may increase the expression of proteasomal subunits, which did occur in our study; given the age-associated reductions in proteostasis network pathway activity, however, this adaptation only serves to aggravate the dysfunction of the protein degradation machinery (Reis-Rodrigues et al., 2012; Pomatto et al., 2017). Noteworthy is the fact that age is a primary risk factor for the appearance of neurodegenerative diseases such as Alzheimer’s and Parkinson’s diseases, both of which are characterized by abnormal proteostasis and accumulation of abnormal protein aggregates (Lim and Yue, 2015).

While our findings are novel and demonstrate the effects of MatSep on individual components of the proteostasis machinery in different brain regions from adult and aged female and male rats, we acknowledge that further studies are required. For instance, although the markers selected for the ALP and mitophagy analyses were aimed at representing distinct stages in these pathways, we recognize that specific and dynamic changes in auto/mitophagy flux induced by ELS may not be detected through our strategy. In addition, transcriptional regulation of autophagy is known to occur in multiple tissues, including the brain (Bernard et al., 2015; Oliván et al., 2015; Di Malta et al., 2019). Determining autophagy marker mRNA levels would be another useful tool in determining MatSep effects. Future studies using an *in situ* approach, such as quantitative confocal microscopy, should be conducted to complement our current investigation. Similarly, in the case of our UPS-related markers, further studies measuring specific protease-like catalytic activities associated with the proteasome will provide further insights into the functional alterations caused by ELS on the UPS. In addition, given that we observed multiple sex-specific effects of ELS, the potential roles of sex hormones should also be addressed in future studies. Lastly, we saw numerous brain region-specific effects of ELS, but we cannot rule out the possibility that ELS may directly or indirectly affect the proteostasis network by influencing other brain regions. It is also important to recognize that the cerebral cortex and the hippocampus are comprised of many subregions, and responses to stress need not necessarily be the same across the entirety of the tissue. Future studies should expand on the number of experimental animals per group and include other brain regions and time points (i.e., adolescence), as well as potential interventions to mitigate the adverse effects of MatSep.

## Conclusion

Our findings indicate that ELS in the form of MatSep can have long–lasting effects on the expression of individual components of the proteostasis machinery, from the neonatal period to adulthood and even into later life. These effects may differentially impact and influence function in distinct brain regions in a sex- and age-specific manner. Here, we demonstrate that normal aging alters the levels of numerous proteostasis marker proteins associated with autophagy, mitophagy, and proteasomal degradation in the hippocampus and cortex of the brain. Moreover, similar alterations were observed in younger adult animals that had experienced a significant stress exposure in early life, suggesting that MatSep could accelerate some aspects of brain aging. Given the well-established relationship between proteostasis dysfunction and neuropathology, disruption of cellular protein degradation pathways by MatSep-induced alterations in proteostasis marker expression could potentially increase vulnerability to neurological diseases in adulthood and with advancing age.

## Data Availability Statement

The original contributions presented in the study are included in the article/Supplementary Material, further inquiries can be directed to the corresponding author.

## Ethics Statement

The animal study was reviewed and approved by Institutional Animal Care and Use Committee of the University of Texas at El Paso (Protocol #A-201006-1).

## Author Contributions

JS-F and KG conceived and designed the study. JS-F, JH, AC, SC, SS, GL, and KG participated in data acquisition, analysis, and interpretation. JS-F and KG wrote the manuscript. All authors contributed to the article and approved the submitted version.

## Conflict of Interest

The authors declare that the research was conducted in the absence of any commercial or financial relationships that could be construed as a potential conflict of interest.
