# Supplementary material for: Neonatal Maternal Separation Modifies Proteostasis Marker Expression in the Adult Hippocampus
Source: Front Mol Neurosci. 2021 Jul 22;14:661993. doi: 10.3389/fnmol.2021.661993 (PMC8383781; doi:10.3389/fnmol.2021.661993)
Supplement: Supplementary file 1 [file Table_1.DOCX]

**Supplementary Table S1.** Primary antibodies used for Western blotting.

| **Antibody** | **Host** | **Dilution** | **Source** | **Catalog** |
| --- | --- | --- | --- | --- |
| Anti-LC3 | Rabbit | 1:500 | Cell Signaling | 3868 |
| Anti-SQSTM1/p62 | Mouse | 1:1000 | Abcam | ab56416 |
| Anti-beclin-1 | Rabbit | 1:1000 | Cell Signaling | 3495 |
| Anti-proteasome 20S alpha+beta | Rabbit | 1:1000 | Abcam | ab22673 |
| Anti-PSMC5 | Rabbit | 1:1000 | Abcam | ab178681 |
| Anti-K48-linkage specific polyubiquitin | Rabbit | 1:500 | Cell Signaling | 8081 |
| Anti-parkin | Mouse | 1:500 | Cell Signaling | 4211 |
| Anti-PINK1 | Rabbit | 1:1000 | Cell Signaling | 6946 |
| Anti-Actin | Mouse | 1:2000 | Thermo Fisher | MA5-11869 |
