# Supplementary material for: Neonatal Maternal Separation Modifies Proteostasis Marker Expression in the Adult Hippocampus
Source: Front Mol Neurosci. 2021 Jul 22;14:661993. doi: 10.3389/fnmol.2021.661993 (PMC8383781; doi:10.3389/fnmol.2021.661993)
Supplement: Supplementary file 2 [file Table_2.DOCX]

**Supplementary Table S2.** Densitometric analysis of proteostasis markers in adult female and male rats. Hippocampal and cortical samples were taken from control animals and those with prior exposure to MatSep, and compared by sex and treatment using two-way ANOVA with Bonferroni post hoc test. The expression of each marker was normalized to actin. Values represent the mean ± standard error of the mean (SEM) of at least three independent blots.

|  |  | | Hippocampus | |  |  |  | | Cortex | |  |  |
| --- | --- | --- | --- | --- | --- | --- | --- | --- | --- | --- | --- | --- |
| Marker | Control Female | Control Male | | MatSep Female | | MatSep Male | Control Female | Control Male | | MatSep Female | | MatSep Male |
| Beclin-1 | 0.244 ± 0.03 | 0.274 ± 0.03 | | 0.339 ± 0.04 | | 0.605 ± 0.07*† | 0.577 ± 0.07 | 0.523 ± 0.05 | | 0.624 ± 0.09 | | 0.619 ± 0.07 |
| LC3-II | 0.751 ± 0.07 | 0.873 ± 0.08 | | 1.032 ± 0.09* | | 1.219 ± 0.14* | 0.551 ± 0.07 | 0.407 ± 0.08 | | 0.761 ± 0.16 | | 0.524 ± 0.09 |
| p62 | 0.691 ± 0.08 | 0.656 ± 0.08 | | 0.997 ± 0.11* | | 1.215 ± 0.11* | 0.763 ± 0.06 | 0.597 ± 0.05 | | 0.783 ± 0.09 | | 0.668 ± 0.05 |
| Parkin | 0.631 ± 0.08 | 0.726 ± 0.09 | | 0.645 ± 0.13 | | 1.222 ± 0.12*† | 1.988 ± 0.35 | 1.750 ± 0.32 | | 1.348 ± 0.34 | | 1.667 ± 0.35 |
| PINK1 | 0.310 ± 0.03 | 0.457 ± 0.05† | | 0.460 ± 0.03* | | 0.289 ± 0.03*† | 0.862 ± 0.17 | 1.034 ± 0.19 | | 0.684 ± 0.19 | | 0.560 ± 0.13 |
| 20S proteasome | 0.328 ± 0.04 | 0.478 ± 0.05† | | 0.518 ± 0.06* | | 0.291 ± 0.04*† | 0.330 ± 0.02 | 0.256 ± 0.03 | | 0.274 ± 0.03 | | 0.288 ± 0.03 |
| PSMC5 | 0.477 ± 0.05 | 0.483 ± 0.07 | | 0.543 ± 0.08 | | 0.670 ± 0.08 | 0.490 ± 0.06 | 0.501 ± 0.04 | | 0.673 ± 0.05* | | 0.534 ± 0.05 |
| K48 pUb proteins | 2.652 ± 0.23 | 3.190 ± 0.54 | | 3.46 ± 0.31 | | 1.737 ± 0.19*† | 3.341 ± 0.41 | 5.393 ± 0.63† | | 4.271 ± 0.75 | | 3.340 ± 0.44* |

**p*≤0.05 when comparing MatSep to same-sex control; †, *p*≤0.05 when comparing between sexes in the same treatment group.
