# Supplementary material for: Neonatal Maternal Separation Modifies Proteostasis Marker Expression in the Adult Hippocampus
Source: Front Mol Neurosci. 2021 Jul 22;14:661993. doi: 10.3389/fnmol.2021.661993 (PMC8383781; doi:10.3389/fnmol.2021.661993)
Supplement: Supplementary file 4 [file Table_4.DOCX]

**Supplementary Table S4.** MatSep and sex differences in proteostasis markers in hippocampus and cortex of adult animals. Degrees of freedom (D.F.), *F*, *t*, and *p* values from two-way ANOVA with Bonferroni post hoc test are shown. A value of *p*≤0.05) is considered to be statistically significant.

|  | Hippocampus | | | | | | | |  | Cortex | | | | | | | |
| --- | --- | --- | --- | --- | --- | --- | --- | --- | --- | --- | --- | --- | --- | --- | --- | --- | --- |
| Marker | D.F. | MatSep | Sex | Matsep  X  Sex | ♀ Con vs ♀ MatSep | ♂ Con vs ♂ MatSep | ♀ Con vs ♂ Con | ♀ MatSep vs ♂ MatSep |  | D.F. | MatSep | Sex | Matsep  X  Sex | ♀ Con vs ♀ MatSep | ♂ Con vs ♂ MatSep | ♀ Con vs ♂ Con | ♀ MatSep vs ♂ MatSep |
| Beclin-1 | 60 | *F*=21.266  *p*<0.001 | *F*=10.36  *p*=0.002 | *F*=6.562  *p*=0.013 | *t*=1.368  *p*=0.176 | *t*=5.416  *p*<0.001 | *t*=0.504  *p*=0.616 | *t*=3.815  *p*<0.001 |  | 56 | *F*=0.926  *p*=0.340 | *F*=0.655  *p*=0.195 | *F*=0.106  *p*=0.746 | *p*>0.05 | *p*>0.05 | *p*>0.05 | *p*>0.05 |
| LC3-II | 72 | *F*=10.364  *p*=0.002 | *F*=2.515  *p*=0.117 | *F*=0.114  *p*=0.737 | *t*=2.002  *p*=0.049 | *t*=2.562  *p*=0.013 | *t*=0.963  *p*=0.339 | *t*=1.263  *p*=0.211 |  | 51 | *F*=2.495  *p*=0.120 | *F*=3.362  *p*=0.072 | *F*=0.201  *p*=0.655 | *p*>0.05 | *p*>0.05 | *p*>0.05 | *p*>0.05 |
| p62 | 62 | *F*=19.868  *p*<0.001 | *F*=0.887  *p*=0.350 | *F*=1.699  *p*=0.197 | *t*=2.116  *p*=0.038 | *t*=4.321  *p*<0.001 | *t*=0.271  *p*=0.787 | *t*=1.506  *p*=0.137 |  | 48 | *F*=0.508  *p*=0.479 | *F*=4.843  *p*=0.032 | *F*=0.158  *p*=0.693 | *t* =0.216  *p*=0.830 | *t* =0.814  *p*=0.419 | *t* =1.904  *p*=0.062 | *t* =1.233  *p*=0.223 |
| Parkin | 59 | *F*=5.426  *p*=0.023 | *F*=9.374  *p*=0.003 | *F*=4.837  *p*=0.032 | *t*=0.0854  *p*=0.932 | *t*=3.494  *p*<0.001 | *t*=0.645  *p*=0.521 | *t*=3.535  *p*<0.001 |  | 44 | *F*=1.036  *p*=0.315 | *F*=0.0125  *p*=0.911 | *F*=0.608  *p*=0.441 | *p*>0.05 | *p*>0.05 | *p*>0.05 | *p*>0.05 |
| PINK1 | 68 | *F*=0.0532  *p*=0.818 | *F*=0.102  *p*=0.75 | *F*=17.885  *p*<0.001 | *t*=2.673  *p*=0.009 | *t*=3.359  *p*=0.001 | *t*=3.065  *p*=0.003 | *t*=2.953  *p*=0.004 |  | 52 | *F*=3.453  *p*=0.068 | *F*=0.0184  *p*=0.893 | *F*=0.715  *p*=0.401 | *p*>0.05 | *p*>0.05 | *p*>0.05 | *p*>0.05 |
| 20S proteasome | 61 | *F*=0.0006  *p*=0.980 | *F*=0.62  *p*=0.434 | *F*=14.88  *p*<0.001 | *t*=2.808  *p*=0.007 | *t*=2.653  *p*=0.01 | *t*=2.298  *p*=0.025 | *t*=3.123  *p*=0.003 |  | 51 | *F*=0.132  *p*=0.718 | *F*=0.926  *p*=0.340 | *F*=1.965  *p*=0.167 | *p*>0.05 | *p*>0.05 | *p*>0.05 | *p*>0.05 |
| PSMC5 | 56 | *F*=3.305  *p*=0.074 | *F*=0.906  *p*=0.345 | *F*=0.769  *p*=0.384 | *p*>0.05 | *p*>0.05 | *p*>0.05 | *p*>0.05 |  | 47 | *F*=4.204  *p*=0.046 | *F*=1.488  *p*=0.228 | *F*=2.045  *p*=0.159 | *t* =2.431  *p*=0.019 | *t* =0.444  *p*=0.659 | *t* =0.150  *p*=0.881 | *t* =1.851  *p*=0.07 |
| K48 pUb proteins | 58 | *F*=0.802  *p*=0.374 | *F*=2.709  *p*=0.105 | *F*=9.842  *p*=0.003 | *t*=1.498  *p*=0.140 | *t*=3.040  *p*=0.004 | *t*=1.124  *p*=0.266 | *t*=3.196  *p*=0.002 |  | 52 | *F*=1.034  *p*=0.314 | *F*=1.029  *p*=0.315 | *F*=7.292  *p*=0.009 | *t*=1.135  *p*=0.261 | *t* =2.771  *p*=0.008 | *t* =2.729  *p*=0.009 | *t* =1.15  *p*=0.255 |
