# Supplementary material for: Neonatal Maternal Separation Modifies Proteostasis Marker Expression in the Adult Hippocampus
Source: Front Mol Neurosci. 2021 Jul 22;14:661993. doi: 10.3389/fnmol.2021.661993 (PMC8383781; doi:10.3389/fnmol.2021.661993)
Supplement: Supplementary file 5 [file Table_5.DOCX]

**Supplementary Table S5.** MatSep and sex differences in proteostasis markers in hippocampus and cortex of aged animals. Degrees of freedom (D.F.), *F*, *t*, and *p* values from one-way ANOVA with Bonferroni post hoc test are shown. A value of *p*≤0.05) is considered to be statistically significant.

|  | Hippocampus | | | | |  | Cortex | | | | |
| --- | --- | --- | --- | --- | --- | --- | --- | --- | --- | --- | --- |
| Marker | D.F. | *F* | *p* | ♀ Con vs ♀ MatSep | ♀ Con vs ♂ Con |  | D.F. | F | p | ♀ Con vs ♀ MatSep | ♀ Con vs ♂ Con |
| Beclin-1 | 38 | 0.86 | 0.431 | *p*>0.05 | *p*>0.05 |  | 39 | 7.607 | 0.002 | *p*>0.05 | *p*=0.005 |
| LC3-II | 38 | 1.65 | 0.204 | *p*>0.05 | *p*>0.05 |  | 33 | 0.252 | 0.779 | *p*>0.05 | *p*>0.05 |
| p62 | 49 | 3.017 | 0.058 | *p*>0.05 | *p*>0.05 |  | 32 | 0.286 | 0.753 | *p*>0.05 | *p*>0.05 |
| Parkin | 33 | 4.803 | 0.015 | *p*>0.05 | *p*>0.05 |  | 38 | 0.097 | 0.907 | *p*>0.05 | *p*>0.05 |
| PINK1 | 38 | 2.754 | 0.076 | *p*>0.05 | *p*>0.05 |  | 36 | 0.846 | 0.438 | *p*>0.05 | *p*>0.05 |
| 20S proteasome | 32 | 0.465 | 0.633 | *p*>0.05 | *p*>0.05 |  | 38 | 1.836 | 0.173 | *p*>0.05 | *p*>0.05 |
| PSMC5 | 38 | 7.996 | 0.001 | *p*=0.017 | *p*=0.002 |  | 35 | 1.344 | 0.274 | *p*>0.05 | *p*>0.05 |
| K48 pUb proteins | 34 | 2.151 | 0.132 | *p*>0.05 | *p*>0.05 |  | 38 | 0.672 | 0.517 | *p*>0.05 | *p*>0.05 |
