# Supplementary material for: Neonatal Maternal Separation Modifies Proteostasis Marker Expression in the Adult Hippocampus
Source: Front Mol Neurosci. 2021 Jul 22;14:661993. doi: 10.3389/fnmol.2021.661993 (PMC8383781; doi:10.3389/fnmol.2021.661993)
Supplement: Supplementary file 6 [file Table_6.DOCX]

**Supplementary Table S6.** MatSep and age differences in the expression of proteostasis markers in adult versus aged hippocampus. Degrees of freedom (D.F.), *F*, and *p* values from one-way ANOVA with Bonferroni post hoc test are shown. A value of *p*≤0.05) is considered to be statistically significant.

|  | Adult MatSep vs Aged Control | | | | |  | | Adult Control vs Aged Control | | | | |
| --- | --- | --- | --- | --- | --- | --- | --- | --- | --- | --- | --- | --- |
| Marker | D.F. | *F* | *p* | Adult ♀ MatSep vs Aged ♀ Con | Adult ♂ MatSep vs Aged ♂ Con |  | D.F. | | *F* | *p* | Adult ♀ Con vs Aged ♀ Con | Adult ♂ Con vs Adult ♂ Con |
| Beclin-1 | 52 | 3.356 | 0.026 | *p*=0.138 | *p*>0.05 |  | 60 | | 14.805 | <0.001 | *p*<0.001 | *p*<0.001 |
| LC3-II | 57 | 17.69 | <0.001 | *p*<0.001 | *p*<0.001 |  | 69 | | 8.862 | <0.001 | *p*=0.006 | *p*=0.012 |
| p62 | 66 | 3.434 | 0.022 | *p*>0.05 | *p*>0.05 |  | 72 | | 5.202 | 0.003 | *p*>0.05 | *p*=0.003 |
| Parkin | 51 | 4.289 | 0.009 | *p*=0.843 | *p*=0.155 |  | 56 | | 7.828 | <0.001 | *p*=0.039 | *p*=0.002 |
| PINK1 | 56 | 5.304 | 0.003 | *p*>0.05 | *p*>0.05 |  | 68 | | 3.786 | 0.014 | *p*=0.072 | *p*=0.302 |
| 20S proteasome | 49 | 1.752 | 0.169 | *p*>0.05 | *p*>0.05 |  | 56 | | 1.03 | 0.386 | *p*>0.05 | *p*>0.05 |
| PSMC5 | 53 | 4.967 | 0.004 | *p*>0.05 | *p*>0.05 |  | 57 | | 7.173 | <0.001 | *p*>0.05 | *p*=0.002 |
| K48 pUb proteins | 50 | 22.74 | <0.001 | *p*=0.071 | *p*<0.001 |  | 56 | | 8.544 | <0.001 | *p*=0.003 | *p*=0.007 |
