# Supplementary material for: Neonatal Maternal Separation Modifies Proteostasis Marker Expression in the Adult Hippocampus
Source: Front Mol Neurosci. 2021 Jul 22;14:661993. doi: 10.3389/fnmol.2021.661993 (PMC8383781; doi:10.3389/fnmol.2021.661993)
Supplement: Supplementary file 7 [file Table_7.DOCX]

**Supplementary Table S7.** MatSep and age differences in the expression of proteostasis markers in adult versus aged cortex. Degrees of freedom (D.F.), *F*, and *p* values from one-way ANOVA with Bonferroni post hoc test are shown. A value of *p*≤0.05) is considered to be statistically significant.

|  | Adult MatSep vs Aged Control | | | | |  | | Adult Control vs Aged Control | | | | |
| --- | --- | --- | --- | --- | --- | --- | --- | --- | --- | --- | --- | --- |
| Marker | D.F. | *F* | *p* | Adult ♀ MatSep vs Aged ♀ Con | Adult ♂ MatSep vs Aged ♂ Con |  | D.F. | | *F* | *p* | Adult ♀ Con vs Aged ♀ Con | Adult ♂ Con vs Adult ♂ Con |
| Beclin-1 | 52 | 9.907 | <0.001 | *p*>0.05 | *p*<0.001 |  | 56 | | 13.85 | <0.001 | *p*=0.832 | *p*<0.001 |
| LC3-II | 51 | 1.635 | 0.193 | *p*>0.05 | *p*>0.05 |  | 53 | | 4.31 | 0.009 | *p*=0.258 | *p*=0.044 |
| p62 | 48 | 8.496 | <0.001 | *p*<0.001 | *p*=0.097 |  | 51 | | 9.472 | <0.001 | *p*<0.001 | *p*=0.517 |
| Parkin | 44 | 7.235 | <0.001 | *p*=0.131 | *p*=0.002 |  | 47 | | 12.158 | <0.001 | *p*<0.001 | *p*=0.004 |
| PINK1 | 52 | 0.305 | 0.821 | *p*>0.05 | *p*>0.05 |  | 55 | | 1.349 | 0.268 | *p*>0.05 | *p*>0.05 |
| 20S proteasome | 51 | 15.03 | <0.001 | *p*<0.001 | *p*<0.001 |  | 54 | | 15.553 | <0.001 | *p*=0.001 | *p*<0.001 |
| PSMC5 | 47 | 6.892 | <0.001 | *p*<0.001 | *p*>0.05 |  | 48 | | 1.483 | 0.231 | *p*>0.05 | *p*>0.05 |
| K48 pUb proteins | 52 | 4.433 | 0.008 | *p*=0.452 | *p*=0.021 |  | 55 | | 4.058 | 0.011 | *p*=0.032 | *p*>0.05 |
