# Supplementary figures and images for: Neonatal Maternal Separation Modifies Proteostasis Marker Expression in the Adult Hippocampus
Source: Front Mol Neurosci. 2021 Jul 22;14:661993. doi: 10.3389/fnmol.2021.661993 (PMC8383781; doi:10.3389/fnmol.2021.661993)

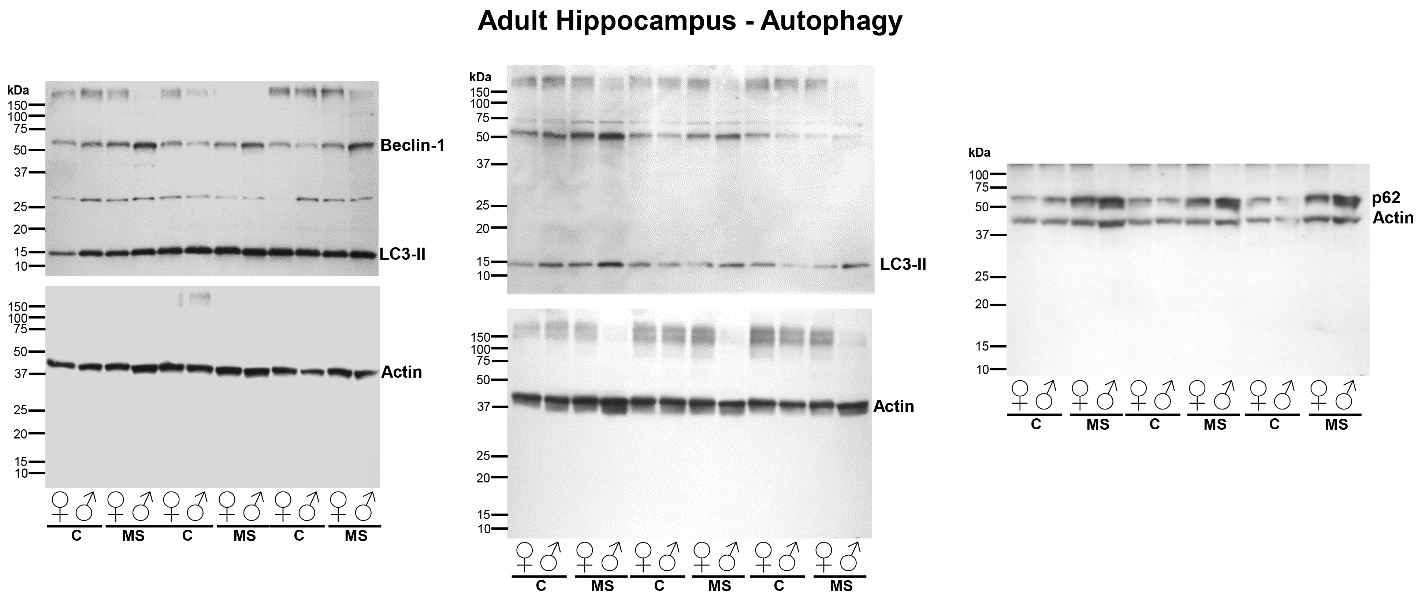


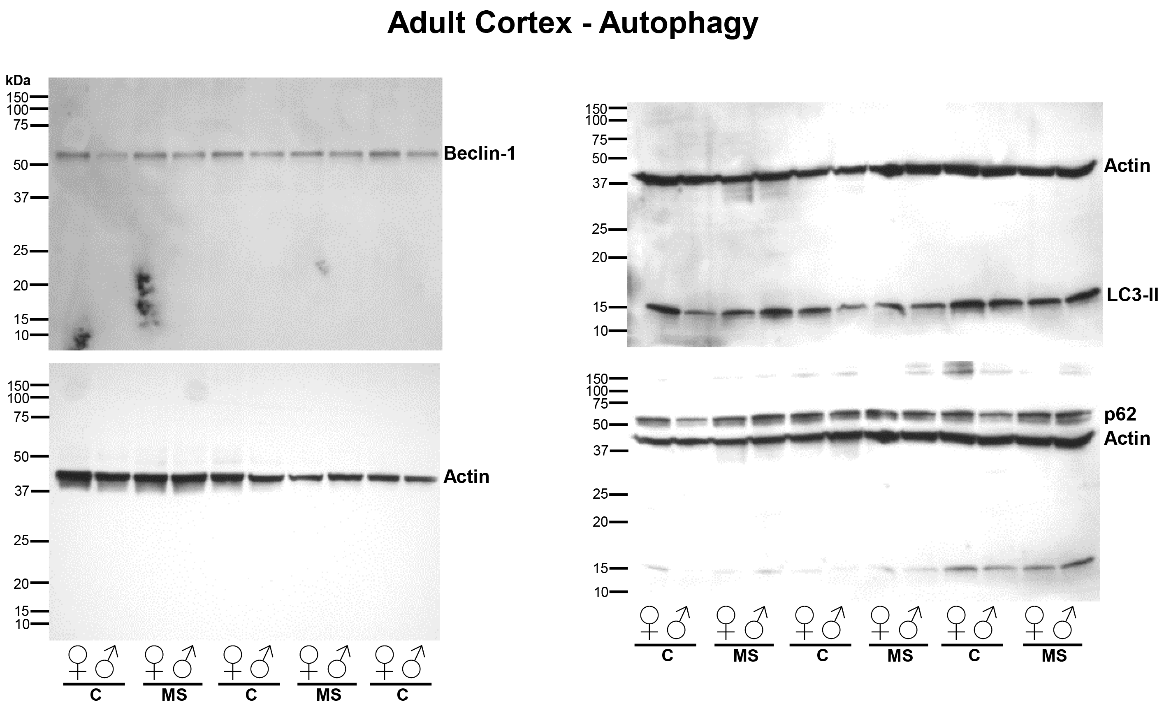


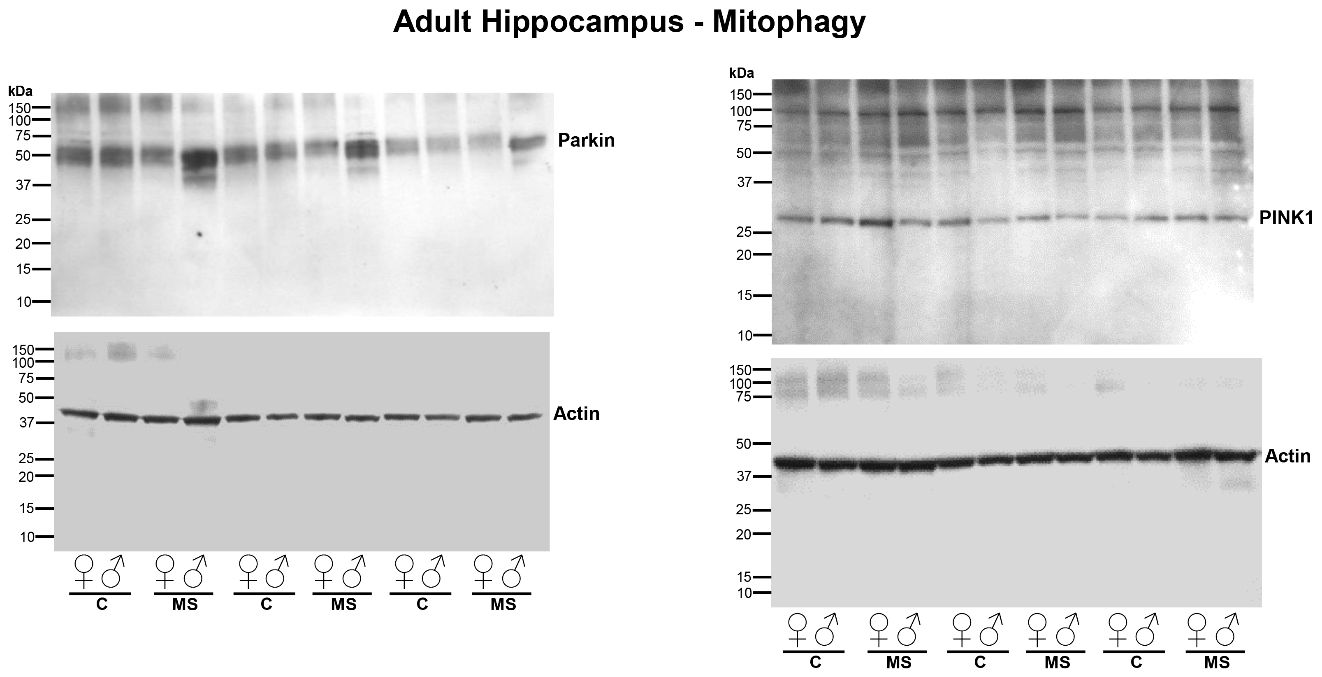


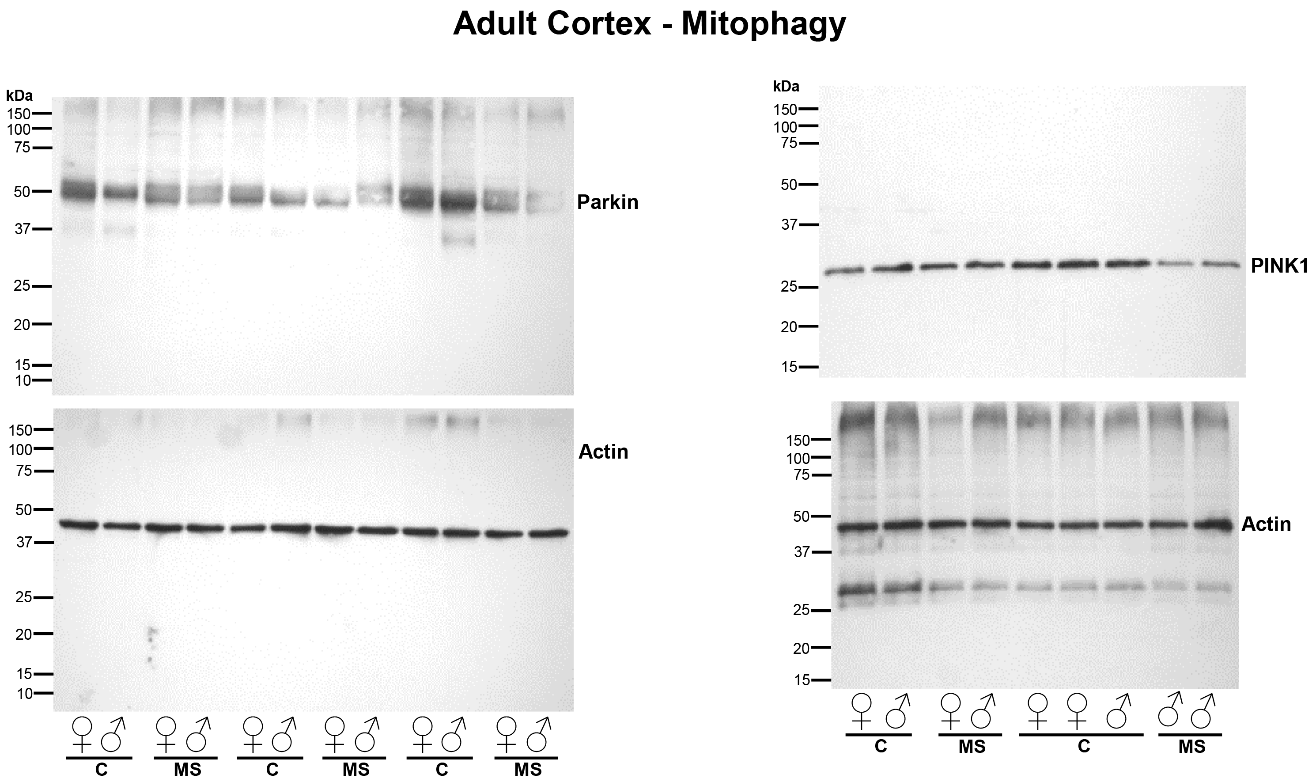


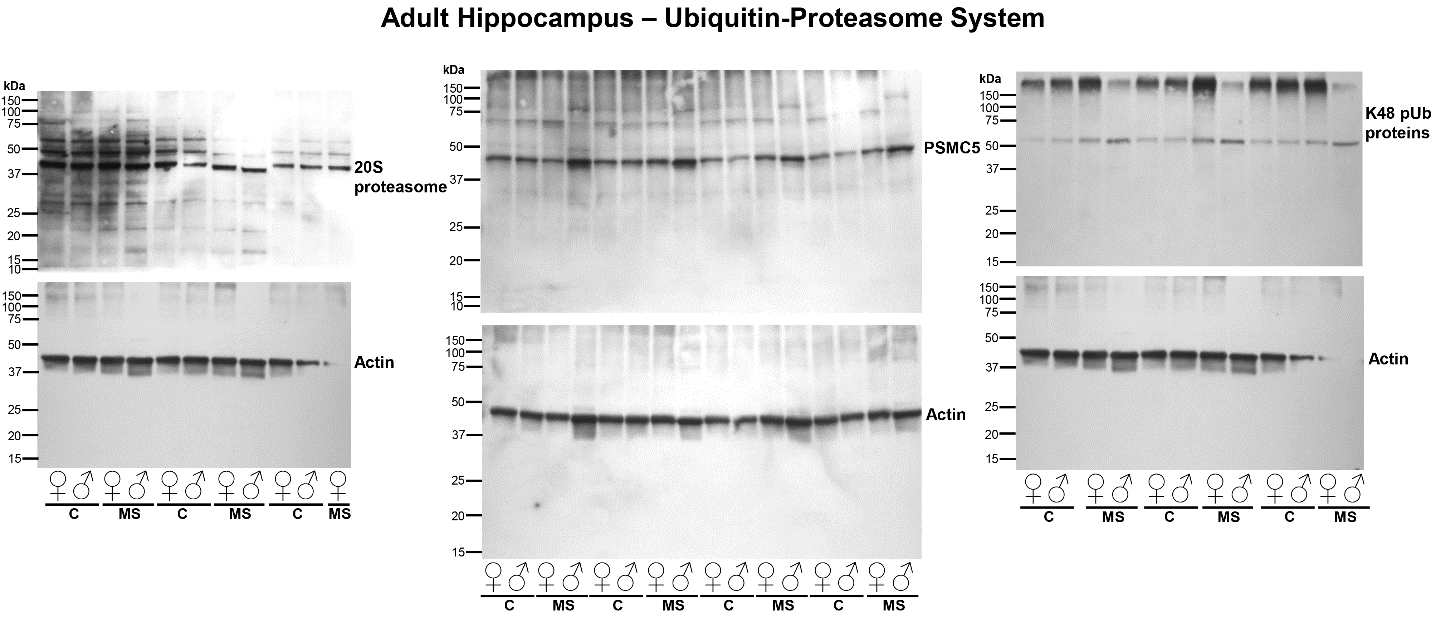


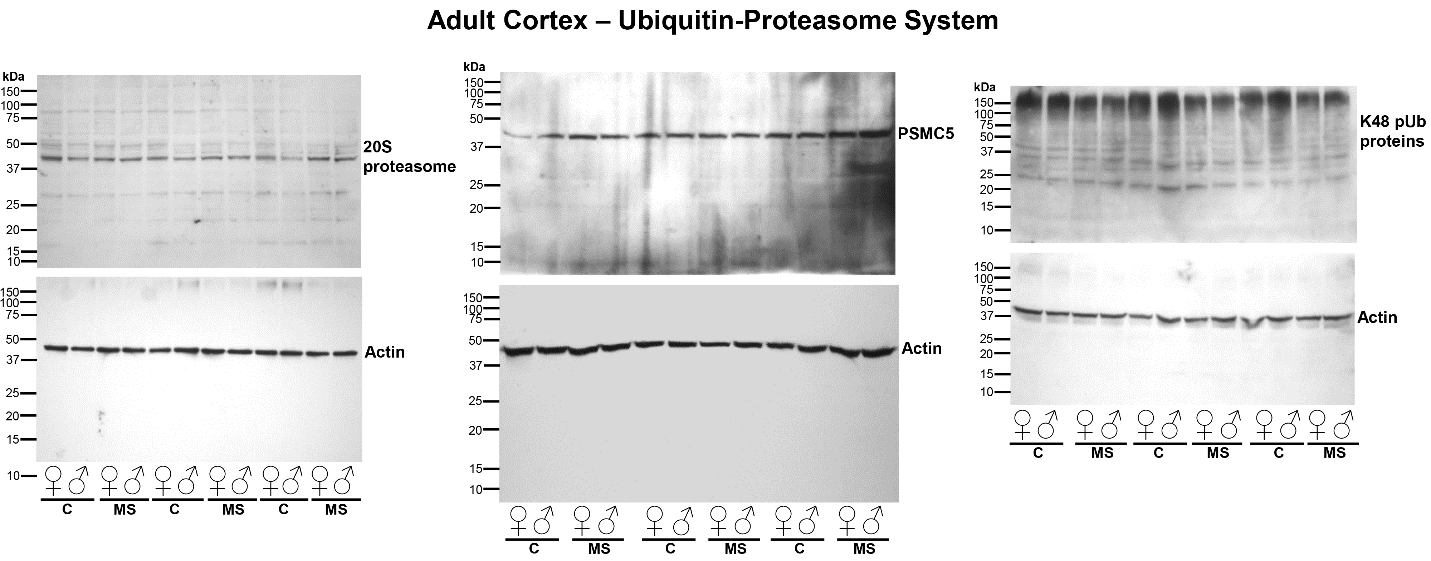


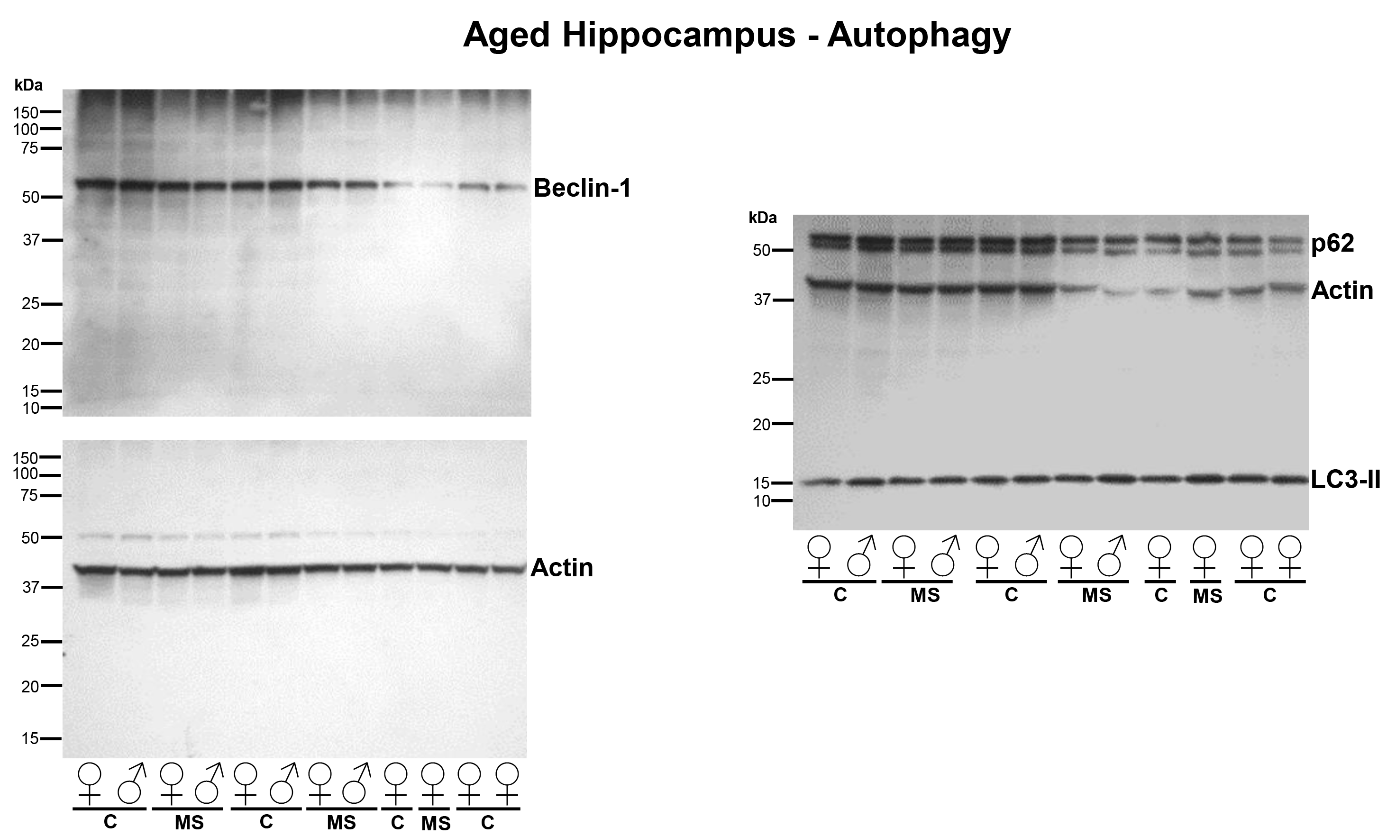


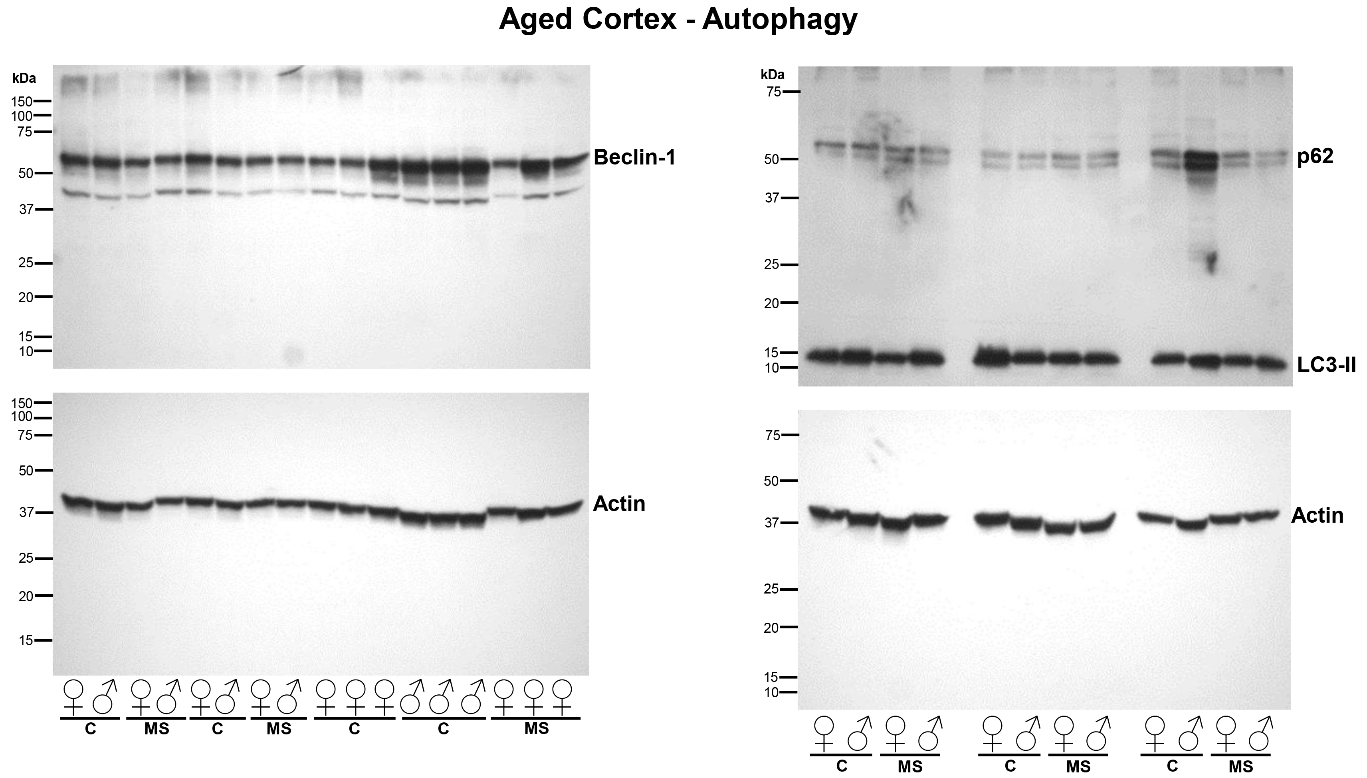


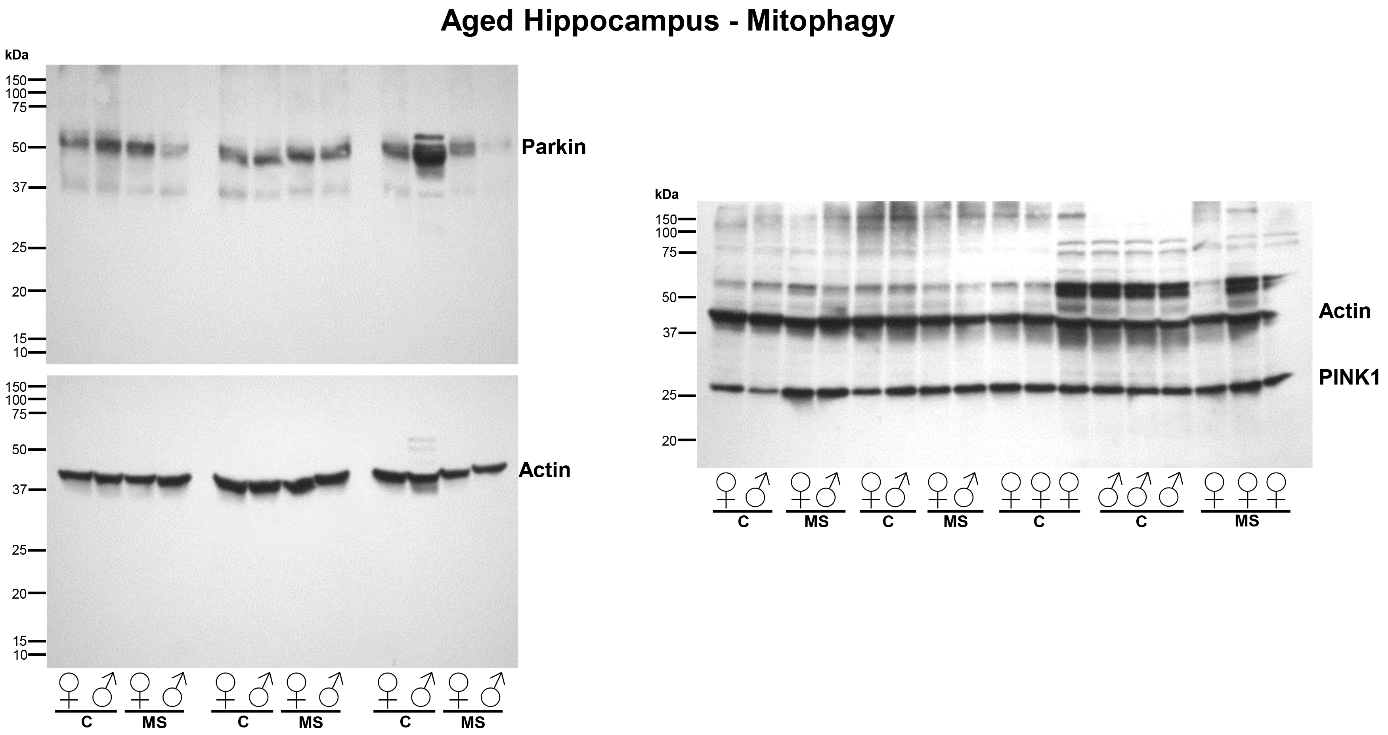


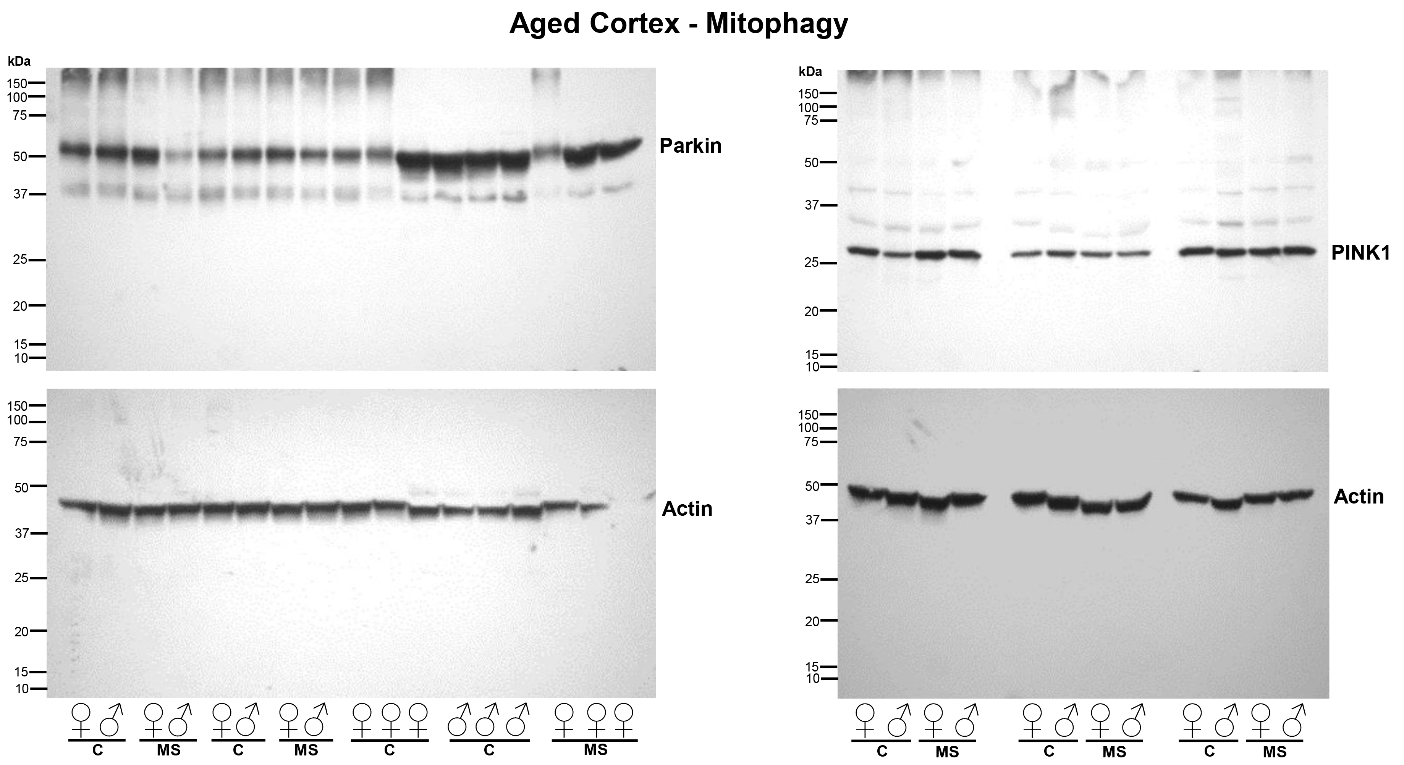


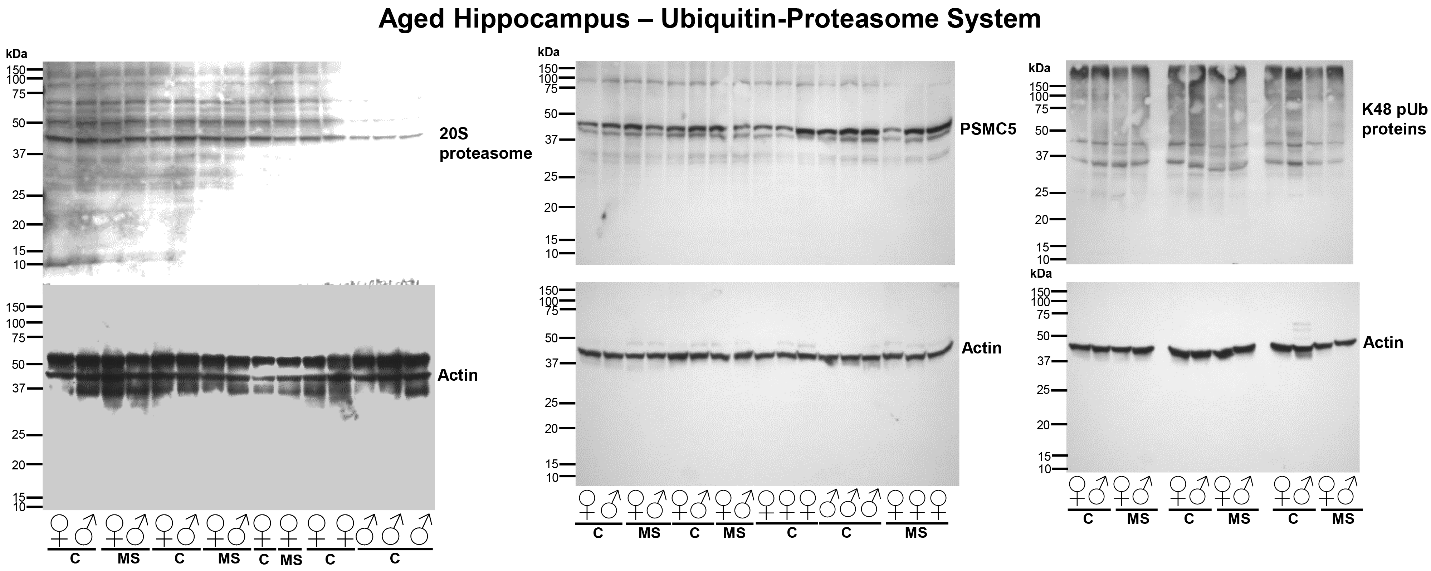


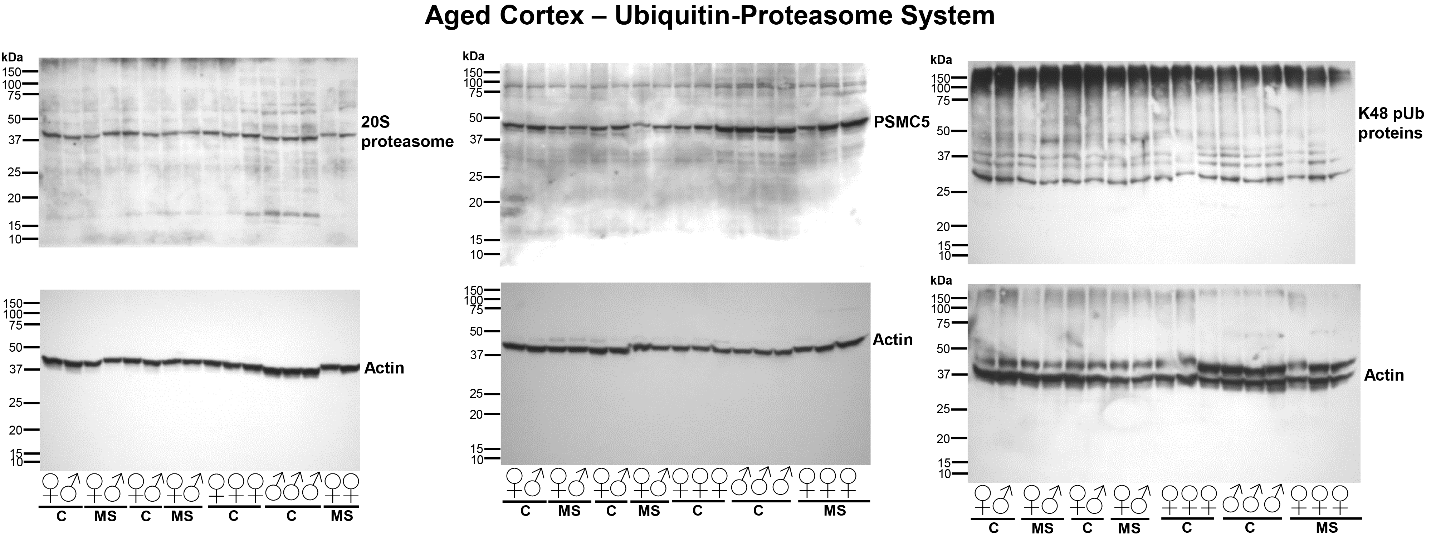

Supplement: Supplementary file 8 [file Data_Sheet_1.docx]
